# What is value—accumulated reward or evidence?

**DOI:** 10.3389/fnbot.2012.00011

**Published:** 2012-11-02

**Authors:** Karl Friston, Rick Adams, Read Montague

**Affiliations:** ^1^Wellcome Trust Centre for Neuroimaging, University College LondonLondon, UK; ^2^Department of Physics, Virginia Tech Carilion Research Institute, Virginia TechRoanoke, VA, USA

**Keywords:** free energy, active inference, value, evidence, surprise, self-organization, selection, Bayesian

## Abstract

Why are you reading this abstract? In some sense, your answer will cast the exercise as valuable—but what is value? In what follows, we suggest that *value is evidence* or, more exactly, log Bayesian evidence. This implies that a sufficient explanation for valuable behavior is the accumulation of evidence for internal models of our world. This contrasts with normative models of optimal control and reinforcement learning, which assume the existence of a value function that explains behavior, where (somewhat tautologically) behavior maximizes value. In this paper, we consider an alternative formulation—active inference—that replaces policies in normative models with prior beliefs about the (future) states agents should occupy. This enables optimal behavior to be cast purely in terms of inference: where agents sample their sensorium to maximize the evidence for their generative model of hidden states in the world, and minimize their uncertainty about those states. Crucially, this formulation resolves the tautology inherent in normative models and allows one to consider how prior beliefs are themselves optimized in a hierarchical setting. We illustrate these points by showing that any optimal policy can be specified with prior beliefs in the context of Bayesian inference. We then show how these prior beliefs are themselves prescribed by an imperative to minimize uncertainty. This formulation explains the saccadic eye movements required to read this text and defines the value of the visual sensations you are soliciting.

## Introduction

So, why are you reading this paper? According to what follows, the answer is fairly simple: you are compelled to selectively sample sensory input that conforms to your predictions and—*a priori*—you believe that reading this text will reduce your uncertainty about what we are going to say (you are going to see) next. This may sound a rather trite explanation but it contains two fundamental premises. Both of these premises can be motivated from the basic principles of self-organization: namely, the imperative to minimize surprise (maximize evidence) associated with sensory states—by actively sampling the environment—and the imperative to minimize uncertainty about the inferred causes of that input—by making inferences about future or fictive states. Together, these provide a complete account of optimal behavior, in which value becomes log-evidence or negative surprise. This paper tries to unpack these assertions using formal arguments and simulations. In fact, the final simulation reproduces a simple form of reading, in which an agent garners evidence for its beliefs using saccadic eye movements (Rayner, 1978).

Implicit in this account of optimal behavior is a hierarchical perspective on optimization, in which behavior is cast as active Bayesian inference that is constrained by prior beliefs. Crucially, these prior beliefs are themselves optimized at a higher hierarchal level. This is important because it resolves the tautology inherent in normative schemes based upon optimal control theory and cost or reward functions. The tautology here is almost self-evident: if behavior is optimal, then it maximizes value. But what is value—other than an objective function that describes optimal behavior. It is this descriptive (circular) aspect of conventional formulations we associate with normative schemes. Put simply, adopting a normative model subverts questions about the origin and optimization of value functions *per se*. For example, it would be difficult to specify a reward or value function that explains why you are reading this text.

In the context of active inference, this issue is resolved by appeal to hierarchical Bayesian inference, in which optimization at one level is constrained by *empirical* priors from a higher level. Optimization in this setting refers to maximizing Bayesian model evidence (or minimizing surprise). In most real-world examples—for example the Bayesian brain (Yuille and Kersten, 2006)—a hierarchical aspect to inference emerges naturally from a separation of temporal scales. For example, inference about the causes of some data is constrained by the parameters of a generative model that are learned after all the data have been seen. Similarly, the form of the model itself can be optimized through model selection, after the parameters of competing models have been optimized. Neurobiologically, these optimization or inference processes may be associated with synaptic activity, synaptic plasticity and synaptic regression—each operating at successively slower timescales. Although the optimization processes may differ (e.g., neuronal dynamics, associative learning, and neurodevelopment), they are all fulfilling the same objective; namely, to maximize the Bayesian model evidence averaged over time. Clearly, one can develop this hierarchical perspective to an evolutionary level, where natural selection may play the role of Bayesian model selection. In short, contextualizing optimization processes at different temporal scales allows one to examine the process theories (putative implementation) at each level and consider them in relation to the level above. We will see an example of this later, in terms of empirical prior beliefs that are updated slowly after fast eye movements. Furthermore, formulating optimal behavior in terms of active inference means that one can associate value in normative schemes with probabilistic attributes of sensory states. This is important because it provides a link between normative models of optimal control and normative models based upon information theory (Barlow, 1961; Linsker, 1990; Bialek et al., 2001; Zetzsche and Röhrbein, 2001)—such as the principle of least action, the principle of maximum entropy, the principle of minimum redundancy and the principle of maximum information transfer. This link rests on replacing reward or cost functions in optimal control theory with prior beliefs in the context of Bayes-optimal inference.

## Overview

This paper comprises six sections. The first three focus on conventional optimal control and reinforcement learning schemes and their formulation in terms of active inference. In particular, they show how cost functions can be replaced by prior beliefs under active inference. These sections use discrete time formulations and summarises the material in Friston et al. (2012b). The final three sections consider where prior beliefs come and move from the abstract formulations of normative models to biophysically realistic formulations. These sections use continuous time and summarises the material in Friston et al. (2012a).

The first section reviews the role of cost and value functions in Markov decision processes (MDPs) and their extensions to partially observable Markov decision processes (POMDPs). We then revisit these formulations from the point of view of active inference and demonstrate their formal relationships. In brief, active inference separates *inference* about hidden states causing observations from *action*. The motivation for this is pragmatic; in that real agents cannot know how their action affects hidden states (because hidden states have to be inferred). This means that action must be based on a function of observed states, as opposed to hidden states. Active inference assumes that this function is the same variational free energy used in approximate Bayesian inference (Hinton and van Camp, 1993; Dayan et al., 1995; MacKay, 1995; Neal and Hinton, 1998). In other words, active inference extends the minimization of variational free energy that underlies approximate Bayesian inference to *include action* (Friston et al., 2010b). However, requiring action to minimize variational free energy appears to contradict optimal control theory, which requires action to minimize expected cost. The purpose of the second section is to resolve this conflict. We will see that the cost functions that are used to guide action in optimal control can be absorbed into prior beliefs in active inference. Effectively, this means that agents expect their state transitions to minimize cost, while action realizes these prior beliefs by maximizing the marginal likelihood of observations. This means one can use standard Bayesian inference schemes to solve optimal control problems—see also McKinstry et al. (2006). The third section illustrates this by showing how optimal policies can be inferred under prior beliefs about future (terminal) states using standard variational Bayesian procedures (Beal, 2003). This section concludes with an example (the mountain car problem) that illustrates how active inference furnishes online nonlinear optimal control, with partially observed (hidden) states.

The fourth section turns to the nature and origin of prior beliefs and shows how they can be derived from the basic imperatives of self-organization (Ashby, 1947; Tschacher and Haken, 2007). This section uses a general but rather abstract formulation of agents—in terms of the states they can occupy—that enables us to explain action, perception and control as corollaries of variational free energy minimization. The focus here is on prior beliefs about control and how they relate to the principle of maximum mutual information and specific treatments of visual attention such as Bayesian surprise (Itti and Baldi, 2009). Having established the underlying theory, the fifth section considers neurobiological implementations in terms of predictive coding and recurrent message passing in the brain. This section reprises a neural architecture we have described in previous publications and extends it to include the encoding of prior beliefs in terms of (place coded) saliency maps. The final section provides an illustration of the basic ideas, using neuronally plausible simulations of visual search and the control of saccadic eye movements. This illustration allows us to understand Bayes-optimal searches in terms of the accumulation of evidence during perceptual synthesis.

## Markovian formulations of value and optimal control

In the following sections, we apply variational free energy minimization to a well-studied problem in optimal decision theory, psychology and machine learning; namely MDPs. In brief, we show that free energy minimization (active inference) and optimal decision theory provide the same solutions when the *policies* from optimal decision theory are replaced by *prior beliefs* about transitions from one state to another. This is important because specifying behavior in terms of prior beliefs finesses the difficult problem of optimizing behavior to access distal rewards. Furthermore, it enables one to consider optimality in terms of accessing particular states in the future. Bayes-optimal behavior then depends upon a representation of future behaviors that necessarily entails a model of agency.

This section considers discrete time (Markov) decision processes of the sort found in optimal control theory, models of behavior and decision making (Bellman, 1952; Watkins and Dayan, 1992; Camerer, 2003; Daw and Doya, 2006; Todorov, 2006; Dayan and Daw, 2008). Its aim is to establish a link between classical approaches to optimizing decisions, in terms of policy optimization, and the variational free energy minimization that underlies active inference (Beal, 2003; Friston et al., 2009). Here, classical schemes are taken to imply that actions (and beliefs about hidden states of the world) are chosen to maximize the expected reward of *future states*. Conversely, in active inference, actions and beliefs minimize a variational free energy bound on the (negative log) marginal likelihood of *observed states*—that is, they maximize the marginal likelihood or Bayesian model evidence. Linking the two formulations necessarily requires us to formulate free energy minimization in discrete time and think about how reward or cost functions are accommodated.

The key distinction between optimal control and active inference is that in optimal control, action optimizes the expected cost associated with the hidden states a system or agent visits. In contrast, active inference requires action to optimize the marginal likelihood (Bayesian model evidence) of observed states, under a generative model. This introduces a distinction between cost-based optimal control and Bayes-optimal control that eschews cost. The two approaches are easily reconciled by ensuring the generative model embodies prior beliefs about state transitions that minimize expected cost. Our purpose is therefore not to propose an alternative implementation of optimal control but accommodate optimal control within the larger framework of active inference.

### Markov decision processes

First, we briefly consider Markov decision problems and their solutions based upon cost or reward functions that are an integral part of optimal control theory and reinforcement learning.

**Notation and set up**: We will use *X* for a finite set of states and *x* ∈ *X* for particular values. A probability distribution will be denoted by *P*(*x*) = Pr {*X* = *x*} using the usual conventions. The tilde notation $\tilde{x}=(x_{0},\;\ldots ,\;x_{T})$ denotes a sequence of values at time points *t* = 0, …, *T*.

**Definition:** A Markov decision process is the tuple (*X*, *A*, *T*, *r*), where

- *Hidden states X*—a finite set of states.
- *Action A*—a finite set of actions.
- *Transition probability T*(*x*′|*x*, *a*) = Pr({*x*_*t*+1_ = *x*′|*x*_*t*_ = *x*, *a*_*t*_ = *a*})—the probability that the state *x*′ ∈ *X* at time *t*+1 follows action *a* ∈ *A* in state *x* ∈ *X* at time *t*.
- *Reward r*(*x*) ∈ ℝ—some reward received at state *x*′ ∈ *X*.

**Problem:** The goal is to find a *policy* π : *X* → *A* that maximizes cumulative rewards. This can be expressed in terms of the sequence of actions ã := (*a*_0_, …, *a*_*T*_) that maximizes *value* or negative *cost-to-go*:

(1) $$V(x)=\underset{\tilde{a}}{\max}\left\{r(x)+\sum_{i=1}^{T}\sum_{x^{\prime }}\mathrm{Pr}(\{x_{i}=x^{\prime }|x_{0}=x,\;a_{0},\ldots ,a_{i}\})r(x^{\prime })\right\}$$

The solution to this equation is a policy or sequence of optimal actions *a*_*t*_: = π(*x*_*t*_) that maximizes expected reward in the future, given a probabilistic model of state transitions. In this setting, (*T*, *r*) constitutes a model that comprises a transition matrix and rewards defined on states. Equation (1) can be expressed as the *Bellman optimality equation* by exploiting the Markovian nature of the problem using recursive substitution (Bellman, 1952):

(2) $$V(x)=\underset{a}{\max}\left\{r(x)+\sum_{s^{\prime }}T(x^{\prime }|x,a)V(x^{\prime })\right\}$$

For simplicity, we have assumed a *finite horizon* problem, in which the reward is maximized from *t* = 0 to *t* = *T*. This allows us to eschew notions of discounting required in infinite horizon problems. Solutions to MDPs can be divided into *reinforcement learning* schemes that compute the value function explicitly and *direct policy searches* that find the optimal policy directly.

In direct policy searches (Williams, 1992; Baxter et al., 2001; Gomez and Miikkulainen, 2001), a policy is optimized by mapping each state directly to an action, without reference to the value of the state. Direct policy searches are useful when the value function is hard to learn but the policy is easy to find. In reinforcement learning there are two general approaches: The first *model based* schemes compute the value function using a model of state transitions and is usually considered when the state space is sufficiently small. This is also known as *dynamic programming* and involves iterating the following two steps (Bellman, 1952):

(3) $$\begin{array}{l} \pi (x)=\underset{a}{\arg\max}\left\{r(x)+\sum_{s^{\prime }}T(x^{\prime }|x,a)V(x^{\prime })\right\}\\ V(x)=r(x)+\sum_{s^{\prime }}T(x^{\prime }|x,\pi (x))V(x^{\prime }) \end{array}$$

This scheme is guaranteed to find the optimal solution, provided all states are visited. In *value iteration* or *backwards induction*, the policy is only calculated when needed. This gives the combined step in (1). In *policy iteration* (Howard, 1960), the first step is repeated until convergence, thereby providing a definite stopping condition. If the transition probabilities or rewards are unknown or the state space is large (precluding a visit to every state), the problem is usually solved with *model free* reinforcement learning. In these schemes the value function is itself learnt (Rescorla and Wagner, 1972; Sutton and Barto, 1981; Watkins and Dayan, 1992; Friston et al., 1994): This enables one to solve Markov decision problems without learning the transition probabilities, because the value function acts as a guidance function for action.

### Partially observable Markov decision processes

The formulation above assumes that the agent knows what state it is in. This is often unrealistic because an agent cannot know the exact state of the world, given noisy or partial observations (Rao, 2010). This leads to an extension of the MDP framework to accommodate partially observed states (Kaelbling et al., 1998); namely a POMDP. Although it is possible to solve POMDPs using direct policy searches (Gomez et al., 2009), one cannot perform value iteration or reinforcement learning directly, as they require the hidden states. However, a POMDP can be converted to a MDP using beliefs about the current state that can be computed recursively from the observations and actions using Bayes rule. This enables one to convert the partially observed process to a (Belief) MDP by treating the beliefs as states and replacing reward with its expected value under the current belief state.

In summary, conventional approaches to MDPs rest on the optimization of future rewards and specify an optimal policy in terms of an action from any given state. Partially observed MDPs make inference explicit by introducing a probabilistic mapping between hidden states of the world and observations. In this setting, the beliefs that the agent forms (by observing histories of actions and states) can be exploited to optimize behavior.

### Optimal control as inference

Our focus is on optimal decision making or control as an inference process: see Filatov and Unbehauen (2004) for a review of early work in this area. Initial approaches were based on converting control problems into inference problems—by replacing reward with an auxiliary random variable conditioned on desired observations. This makes maximizing reward equivalent to maximizing the likelihood of desired observations (Cooper, 1988; Shachter, 1988). Subsequent work focused on efficient methods to solve the ensuing inference problem (Jensen et al., 1994; Zhang, 1998). Later, Dayan and Hinton (1997) proposed an Expectation Maximization algorithm for reinforcement learning with immediate rewards, while Toussaint and Storkey (2006) cast the problem of computing optimal policies as a likelihood maximization problem. This generalized the work of Cooper (1988) and Shachter (1988) to the case of infinite horizons and cost functions over future states. More recently, this approach has been pursued by applying Bayesian procedures to problems of optimal decision making in MDPs (Botvinick and An, 2008; Toussaint et al., 2008; Hoffman et al., 2009).

Related work on stochastic optimal control (Kappen, 2005a,b; van den Broek et al., 2008; Rawlik et al., 2010) exploits the reduction of control problems to inference problems by appealing to variational techniques to provide efficient and computationally tractable solutions. In particular, formulating the problem in terms of Kullback–Leibler minimization (Kappen, 2005a,b) and path integrals of cost functions (Theodorou et al., 2010; Braun et al., 2011).

The variational formalism has also found a powerful application in the setting of optimal control and the construction of adaptive agents. For example, Ortega and Braun (2010), consider the problem of optimizing active agents, where past actions need to be treated as causal interventions. They show that that the solution to this variational problem is given by a stochastic controller called the Bayesian control rule, which implements adaptive behavior as a mixture of experts. This work illustrates the close connections between minimizing (relative) entropy and the ensuing active Bayesian inference that we will appeal to the later.

### Summary

In summary, current approaches to partially observed MDPs and stochastic optimal control minimize cumulative cost using the same procedures employed by maximum likelihood and approximate Bayesian inference schemes. Indeed, the formal equivalence between optimal control and estimation was acknowledged by Kalman at the inception of Bayesian filtering schemes (Todorov, 2008). In the next section, we revisit this equivalence and show that any optimal control problem can be formulated as a Bayesian inference problem, within the active inference framework. The key aspect of this formulation is that action does not minimize cumulative cost but maximizes the marginal likelihood of observations, under a generative model that entails an optimal policy.

## Active inference

This section introduces active inference, in which the optimization of action and beliefs about hidden states are treated as two separate processes that both maximize Bayesian model evidence or the marginal likelihood of observations. In active inference, action elicits *observations* that are the most plausible under beliefs about (future) states. This is in contrast to conventional formulations, in which actions are chosen to elicit (valuable) states. We will see that active inference can implement any optimal policy; however, it does not solve the optimal control problem explicitly, because active inference does not minimize cost-to-go but minimizes the surprise of observations (maximizes their marginal likelihood). This follows from the fact that active inference is a corollary of the free energy principle:

### The free-energy principle

The free-energy principle (Friston et al., 2006) tries to explain how agents occupy a small number of attracting states by minimizing the Shannon entropy of the probability distribution over their sensory states. Under ergodic assumptions, this entropy is (almost surely) the long-term time average of self-information or surprise (Birkhoff, 1931). Surprise, or more precisely *surprisal*, is a (probability) measure −ln *P*(*s*_*t*_|*m*) on the states that are sampled by an agent.

Minimizing the long-term average *E*_*t*_[−ln *P*(*s*_*t*_|*m*)] is assured when agents minimize surprise at each time point. Crucially, surprise is just the negative marginal likelihood or Bayesian model evidence, which means minimizing surprise maximizes Bayesian model evidence. Surprise is minimized—approximately or exactly—if agents minimize a variational free energy bound on surprise (Feynman, 1972; Hinton and van Camp, 1993), given a generative model *m* of state transitions (Dayan et al., 1995; Friston, 2010). We will return to the relationship between entropy, surprise and Bayesian model evidence in Section “Bayes-optimal control without cost functions,” when we examine the motivation for free energy minimization in more detail. Here, we consider the nature of active inference in terms of free energy minimization, where free energy is defined in relation to the following definitions:

**Definition**: Active inference rests on the tuple (*X*, *A*, ϑ, *P*, *Q*, *R*, *S*) comprising:

- A finite set of *hidden states X*
- Real valued *hidden parameters* ϑ ∈ ℝ^*d*^
- A finite set of *sensory states S*
- A finite set of *actions A*
- Real valued *internal states* μ ∈ ℝ^*d*^ that parameterize a conditional density
- A *sampling probability R*(*s*′|*s*, *a*) = Pr({*s*_*t*+1_ = *s*′|*s*_*t*_ = *s*, *a*_*t*_ = *a*}) that observation *s*′ ∈ *S* at time *t* + 1 follows action *a* ∈ *A*, given observation *s* ∈ *S* at time *t*
- A *generative probability* $P(\tilde{s},\;\tilde{x},\;\theta |m)=\mathrm{Pr}(\{s_{0},\;\ldots ,\;s_{t}\}=\tilde{s},\;\{x_{0},\;\ldots ,\;x_{T}\}=\tilde{x},\;\vartheta =\theta )$ over observations to time *t*, states at all times and parameters
- A *conditional probability* $Q(\tilde{x},\;\theta |\mu )=\mathrm{Pr}(\{x_{0},\;\ldots ,\;x_{T}\}=\tilde{x},\;\vartheta =\theta )$ over a sequence of states and parameters, with sufficient statistics μ ∈ ℝ^*d*^

**Remarks**: Here, *m* denotes the form of a generative model or probability distribution over sensory and hidden states and parameters: $P_{m}(\tilde{s},\;\tilde{x},\;\theta ):=P(\tilde{s},\;\tilde{x},\;\theta |m)$. For clarity, we will omit the conditioning on *m* for all but prior terms in the generative probability. The sufficient statistics of the conditional probability $Q_{\mu }(\tilde{x},\;\theta ):=Q(\tilde{x},\;\theta |\mu )$ encode a probability distribution over a sequence of hidden states $\tilde{x}=\{x_{0},\;\ldots ,\;x_{T}\}$ and the parameters of the model θ ∈ ϑ. Crucially, the conditional probability and its sufficient statistics encode hidden states in the future and past, which themselves can change with time: for example, μ_*k*_ = {μ^*k*^_0_, …, μ^*k*^_*T*_}, where μ^*k*^_*t*_ is the probability over hidden states at time *t* in the future or past, under the conditional probability at the present time *k*.

The probabilities above (*P*, *Q*, *R*) underwrite the action and perception of the agent—they correspond to its formal beliefs about the sensory consequences of action (sampling probability) and the hidden states causing observations (generative probability). Because the true states generating observations are unknown and unknowable from the point of view of the agent, they can only be inferred in terms of an approximate posterior probability (conditional probability).

There are three important distinctions between this setup and that used by MDPs. As in partially observed MDPs, there is a distinction between states and observations. However, the transition probability over hidden states no longer depends on action. In other words, the agent does not need to know the effect of its actions on the (hidden) state of the world. It is instead equipped with a probabilistic mapping between its actions and their direct sensory consequences—this is the sampling probability. This is a central tenet of active inference, which separates knowledge about the sensory consequences of action from beliefs about the causes of those consequences. In other words, the agent knows that if it moves it will sense movement (c.f. proprioception); however, beliefs about hidden states in the world causing movement have to be inferred. These hidden states may or may not include its own action: the key distinction between the *agency free* and *agency based* schemes considered below depends on whether the agent represents its own action or not.

The second distinction is that hidden states include future and past states. In other words, the agent represents a sequence or trajectory over states. This enables inference about a particular state in the future to change with time. This will become important when we consider planning and agency. Finally, there are no reward or cost functions. This reflects the fact that active inference does not call upon the notion of reward to optimize behavior—optimal behavior minimizes variational free energy, which is a functional of observations and the conditional probability distribution or its sufficient statistics. As we will see below, cost functions are replaced by priors over hidden states and transitions, such that costly states are surprising and are avoided by action.

### Perception and action

The free energy principle states that the sufficient statistics of the conditional probability and action minimize free energy

(4) $$\begin{array}{l} \mu_{t}=\underset{\mu }{\arg\min}F(\{s_{0},\ldots ,s_{t}\},\mu )\\ a_{t}=\underset{a}{\arg\min}\sum_{S}R(s_{t+1}|s_{t},a)F(\{s_{0},\ldots ,s_{t+1}\},\mu_{t}) \end{array}$$

This dual optimization is usually portrayed in terms of perception and action, by associating the sufficient statistics with internal states of the agent (such as neuronal activity) and associating action with the state of effectors or the motor plant. Equation (4) just says that internal states minimize the free energy of currently observed states, while action selects the next observation that, on average, has the smallest free energy. By factorizing the generative probability $P\left(\tilde{s},\;\tilde{x},\;\theta |m\right)=P(\tilde{s}|\tilde{x},\;\theta )P(\tilde{x},\;\theta |m)$ into likelihood and prior probabilities, one can express the free energy as follows:

(5) $$\begin{array}{l} F(\tilde{s},\mu )=E_{Q}[-\ln P(\tilde{s},\tilde{x},\theta |m)]-E_{Q}[-\ln Q(\tilde{x},\theta |\mu )]\\ \;=D_{KL}[Q(\tilde{x},\theta |\mu )||P(\tilde{x},\theta |\tilde{s})]-\ln P(\tilde{s}|m) \end{array}$$

The first equality in Equation (5) expresses free energy as a Gibbs energy (expected under the conditional distribution) minus the entropy of the conditional distribution. The second shows that free energy is an upper bound on surprise, because the first (Kullback–Leibler divergence) term is nonnegative by Gibbs inequality (Beal, 2003). This means that when free energy is minimized, the conditional distribution approximates the posterior distribution $Q(\tilde{x},\;\theta |\mu )\approx P(\tilde{x},\;\theta |\tilde{s})$ over hidden states and parameters. This formalizes the notion of unconscious inference in perception (Helmholtz, 1866/1962; Dayan et al., 1995; Dayan and Hinton, 1997) and, under some simplifying assumptions, corresponds to predictive coding (Rao and Ballard, 1999).

This formulation highlights the fact that action selects observable states (not hidden states) that are the least surprising or have the smallest free energy. The free energy is determined by the sufficient statistics of the conditional distribution. The optimization of these sufficient statistics or internal states—the first equality in Equation (4)—rests upon the generative model and therefore depends on prior beliefs. It is these beliefs that specify what is surprising and reproduces the optimal policies considered above. There are clearly many ways to specify the generative probability. We will consider two forms, both of which respect the Markov property of decision processes. The first reproduces the behavior under the optimal policy for Markov decision problems and can be regarded as the corresponding free energy formulation:

### An agency free formulation of optimal policies

The natural generative model for a partially observable Markov decision process can be expressed in terms of a likelihood plus priors over states and parameters, with the following forms:

(6) $$\begin{array}{l} \;P\left(\tilde{s},\tilde{x},\theta |m\right)=P(\tilde{s}|\tilde{x},\theta )P(\tilde{x}|\theta )P(\theta |m)\\ P\left(\{s_{0},\ldots ,s_{t}\}|\tilde{x},\theta \right)=P(s_{0}|x_{0})P(s_{1}|x_{1})\ldots P(s_{t}|x_{t})\\ \;P\left(\tilde{x}|\theta \right)=P(x_{0}|m)\prod_{t=0}^{T-1}P(x_{t+1}|x_{t},\theta ) \end{array}$$

This implies that the current observation depends only on the current hidden state (like a belief MDP), where the hidden states are a Markov process, whose transition probabilities depend upon the parameters (unlike a belief MDP). We will assume that the priors over the parameters *P*(θ|*m*) = δ(θ −θ_π_) make the priors over state transitions equivalent to the optimal policy of the previous section. In other words, we assume the priors have a point mass over values that render the transition probabilities *P*(*x*_*t*+1_|*x*_*t*_, θ_π_) = *T*(*x*_*t*+1_|*x*_*t*_, π(*x*_*t*_)) optimal in the conventional sense. The second equality in Equation (5) shows that minimizing the free-energy, with respect to the sufficient statistics of the conditional distribution, renders it the posterior over hidden states and parameters. This means that the conditional distribution becomes the posterior distribution, where (noting that the posterior and prior over parameters are the same Dirac delta function)

(7) $$Q(\tilde{x},\theta |\mu_{t})\approx P(\tilde{x}|\{s_{0},\ldots ,s_{t}\},\theta )\delta (\theta -\theta_{\pi })$$

We have used an approximate equality here because we are assuming approximate Bayesian inference. In this context, free-energy minimization with respect to action becomes, from Equations (4) and (5):

(8) $$\begin{array}{l} a_{t}=\underset{a}{\arg\min}\sum_{S}R(s_{t+1}|s_{t},a)F(\{s_{0},\ldots ,s_{t+1}\},\mu_{t})\\ \;=\underset{a}{\arg\max}\sum_{S}R(s_{t+1}|s_{t},a)E_{Q(x_{t+1})}[\ln P(s_{t+1}|x_{t+1})]\\ Q(x_{t+1})\approx \sum_{X}P(x_{t+1}|x_{t},\pi (x_{t}))P(x_{t}|\{s_{0},\ldots ,s_{t}\}) \end{array}$$

Note that the free energy of the new observation is just its improbability, expected under posterior beliefs about the hidden states that cause it—these posterior beliefs correspond to the marginal conditional distribution *Q*(*s*_*t* + 1_), over the next hidden state.

It can be seen from Equation (8) that action under active inference is exactly the same as action under the optimal policy. This is because action selects the observation that is most likely under the (approximate) posterior distribution. In turn, this is the hidden state that follows the currently inferred state, under the optimal policy. This means that active inference can be considered as a generalization of optimal control. This is because there are prior beliefs that can reproduce an optimal policy to minimize expected cost. However, there are prior beliefs that specify Bayes-optimal control that cannot be expressed as minimizing value (Friston and Ao, 2012). Put simply, although prior beliefs about a particular trajectory through state space may be the solution to an optimal control problem, there may be prior beliefs that are not. These prior beliefs are particularly relevant in robotics and the continuous time formulations considered later. In brief, any trajectory specified by a prior belief can be decomposed into divergence and curl free components (by the fundamental theorem of vector calculus or the Helmholtz decomposition). Crucially, only the curl free (irrotational) component can be specified by a value function. This is problematic because nearly every real-world movement trajectory has divergence free components; such as the rotational components of walking, reading and writing. These are relatively easy to specify and simulate using appropriate priors—see the handwriting simulations in Friston et al. (2011) or the animate behaviors in Tani (2003)—but cannot be specified in terms of a value function of states. See Friston and Ao (2012) for a technical discussion and Friston (2011) for a discussion in the setting of motor control.

### Summary

In summary, we have seen that is fairly straightforward to place optimal decision or Markovian control theory schemes in an active inference framework. This involves replacing optimal policies, defined by cost or reward functions, with prior beliefs about transitions among hidden states. The advantage of doing this is that we can formulate action and perception as jointly minimizing the same objective function that provides an upper bound on surprise or negative log Bayesian evidence. This enables optimal control to be cast as Bayesian inference, with a clear distinction between action and inference about partially observed or hidden states. We will see later that formulating the optimal control problem in terms of prior beliefs enables us to connect to other normative theories about perception and entertain questions about where these prior beliefs come from. For example, the prior beliefs above depend upon the parameters of the generative model (transition probabilities among hidden states) that can be learned in a Bayes-optimal sense. See Friston et al. (2009) for an example.

The fact that one can replace cost functions with priors to produce the same behavior is related to the complete class theorem (Brown, 1981). The complete class theorem states that any admissible decision rule (behavior) is Bayes-optimal for at least one pair of prior beliefs and cost function (Robert, 1992). However, this pair is not necessarily unique: in other words, the same decisions can be reproduced under different combinations of prior and cost functions. In one sense, this duality is resolved by replacing the cost functions of optimal control theory with prior beliefs about state transitions. Casting Bayes-optimal decisions in this way simply means that the agent believes it will sample state space in a way that minimizes future costs, while action fulfills these prior beliefs. In the next section, we consider what would happen if the agent inferred its own action:

## Bayes-optimal control without cost functions

In this section, we consider agency based optimization, in which the hidden states are extended to include hidden (control), states that model action. This is necessary, when inferring optimal state transitions, because transitions depend upon action in the future which is hidden from observation. In what follows, we focus on policies that are specified by prior beliefs about specific states that will be occupied at specific times in the future. This corresponds to a finite horizon control problem with terminal costs over states and intermediate control costs that are specified through prior beliefs about control.

### Agency-based optimization

In what follows, we describe a scheme for POMDPs that optimizes action in relation to prior beliefs about future states. This scheme uses representations of hidden states in the future to optimize a sequence of fictive actions before they are enacted. This calls for a more sophisticated generative model—a model of agency or control. In other words, the agent must infer its future actions via Bayesian updates of posterior beliefs about the future. The heuristic benefit of introducing hidden control states is that future actions can be optimized, when choosing the best current action. The ensuing solutions are optimal in relation to prior beliefs about states that will be occupied. These are prior beliefs about the final (desired) hidden state and can be expressed in terms of the following generative model:

**An agency based model**: The generative probability used in this section introduces (a finite set of) hidden control states *u* ∈ *U* and can be expressed in terms of the following likelihood and prior distributions:

(9) $$\begin{array}{l} \;P\left(\tilde{s},\tilde{x},\tilde{u},\theta |m\right)=P(\tilde{s}|\tilde{x},\theta )P(\tilde{x},\tilde{u}|\theta )P(\theta |m)\\ P\left(\{s_{0},\ldots ,s_{t}\}|\tilde{x},\theta \right)=P(s_{0}|x_{0},\theta )P(s_{1}|x_{1},\theta )\ldots P(s_{t}|x_{t},\theta )\\ \;P\left(\tilde{x},\tilde{u}|\theta \right)=P\text{​}\left(x_{T}|\theta \right)\prod_{t=1}^{T}P\left(x_{t-1}|x_{t},u_{t},\theta \right)\text{​}P\text{​}\left(u_{t}|\theta \right) \end{array}$$

**Remarks**: There are two important aspects of this generative model: First, control states are not action—they are an internal representation of action that may or may not be related to actions emitted by the agent. In the generative model, control states affect the transitions among hidden states; in other words, they only affect outcomes vicariously through hidden states. It is these control states that represent agency, which may or may not be a veridical representation of what the agent can actually do (or is doing)—in this sense, they can be regarded as fictive action that gives the generative model extra degrees of freedom to model state transitions under prior beliefs. Recall that action only changes observations and is selected on the basis of posterior beliefs about the next observable state. Conversely, control states are modeled as hidden states over time and are inferred. This means they only exist in the mind (posterior beliefs) of the agent.

**Figure 1:**
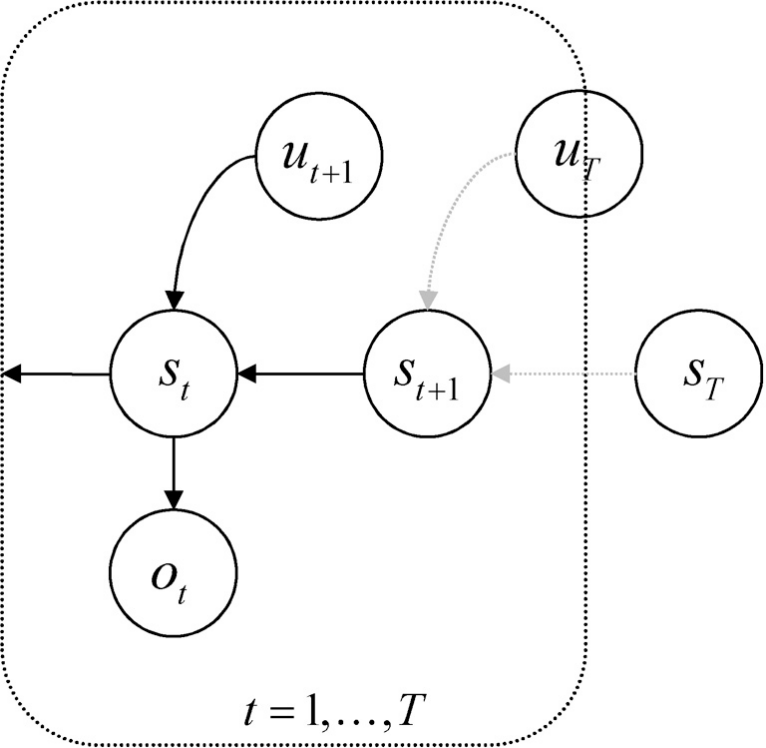
**Probabilistic graphical model illustrating the Markovian dependencies among hidden states generating sensory data.** These hidden states (*s*_*t*_, *u*_*t*_) are represented explicitly, over all time points: *t* = 0, …, *t*. This means there is a representation of the past and future that includes hidden states mediating control. Note that the dependency of this hidden Markov model runs backwards in time so that all preceding hidden states are conditioned recursively on the final or terminal (goal) state.

Second, the priors on the hidden states $P(\tilde{x},\;\tilde{u}|\theta )$ are formulated in a pullback sense; that is, they run backwards in time. This preserves the Markov dependencies but allows us to specify the prior over a sequence of states in terms of transition probabilities and a prior distribution over the final (terminal) state. Put simply, the parameters of the (transition) model encode where I came from, not where I am going. See Figure 1. This particular form of prior belief is chosen for convenience, because it accommodates beliefs about the desired final state—of the sort that would be specified with a terminal cost function, *r*(*x*_*T*_).

The generative model in Equation (9) is fairly general and makes no specific assumptions about the implicit cost of inferred control (it does not assume quadratic control costs) or allowable state transitions. In what follows, we illustrate inference or model inversion using a particular parameterization and variational inversion scheme. This example is used to illustrate agency-based inference, accepting that there are many different model parameterizations and inversion schemes that could have been used.

**Generative probability:** The generative model used below comprises the following likelihood and prior distributions:

(10) $$\begin{array}{l} \;P\left(s_{t}|x_{t},\theta \right)=A\cdot x_{t}\\ P(x_{t-1}|x_{t},u_{t},\theta )=\left(\prod_{i}B_{i}^{u_{ti}}\right)\cdot x_{t}\\ \;P(x_{T}|\theta )=c\\ \;P(u_{t}|\theta )=\prod_{i}d_{i}^{u_{ti}} \end{array}$$

The parameters θ = {**A**, **B**_1_, **B**_2_, …, **c**, **d**} of this model are

(11) $$\begin{array}{l} \;A=\{a_{ij}\}:\sum_{j}a_{ij}=1,\;\forall i\\ B_{k}=\{b_{ijk}\}:\sum_{j}b_{ijk}=1,\;\forall i,k\\ \;c=\{c_{i}\}:\sum_{i}c_{i}=1\\ \;d=\{d_{i}\}:\sum_{i}d_{i}=1 \end{array}$$

The parameters in the matrices **B**_*k*_ encode transition probabilities among hidden states that are engaged when the control state *u*_*k*_ = 1, where the control states have a multinomial distribution—only one can be “on” at any time. The hidden states cause observed states through the mapping encoded by **A**. The vectors **c** and **d** encode the prior distribution over the final hidden state and control states, respectively; these specify the goal and prior costs on control.

**Conditional probability**: To exploit the Markovian form of the generative model we will use an efficient approximate inference scheme afforded by variational Bayesian learning (Beal, 2003); for a tutorial see Fox and Roberts (2011). The efficiency rests on replacing posterior dependencies among hidden states (over time) with mean field effects on the marginal probabilities at each time point. This is achieved using the following *mean-field assumption* for the conditional distribution:

(12) $$\begin{array}{l} \;Q(s,u)=\prod_{t=1}^{T}Q(s_{t})Q(u_{t})\\ Q(s_{t}|\alpha_{t})=\prod_{i}\alpha_{ti}^{s_{i}}:\sum_{i}\alpha_{ti}=1\\ Q(u_{t}|\beta_{t})=\prod_{i}\beta_{ti}^{u_{i}}:\sum_{i}\beta_{ti}=1 \end{array}$$

Standard variational Bayesian learning now provides a recipe for optimizing the sufficient statistics (α_*t*_, β_*t*_) of the conditional probability over hidden and control states. The ensuing variational updates for the sufficient statistics μ_*k*_ = {α^*k*^_0_, …, α^*k*^_*t*_, β^*k*^_0_, …, β^*k*^_*t*_} at successive times *k* are Friston et al. (2012b): for *k* = 1 to T until · convergence:

(13) $$\begin{array}{l} \text{for }t=(T-1)\text{ to }(k+1)\\ \;{\alpha^{\prime }}_{t}=\exp([\ln A^{T}\cdot s_{t}]+\sum_{j}\beta_{(t+1)j}^{k}\ln B_{j}\cdot \alpha_{\left(t+1\right)}^{k}+\sum_{j}\beta_{tj}^{k}\ln B_{j}^{T}\cdot \alpha_{\left(t-1\right)}^{k})\\ \alpha_{t}^{k+1}=\frac{{\alpha^{\prime }}_{t}}{\sum_{i}{\alpha^{\prime }}_{ti}}\\ \;{\beta^{\prime }}_{ti}=\exp(\alpha_{t-1}^{kT}\cdot \ln B_{i}\cdot \alpha_{t}^{k}+\ln d_{i})\\ \beta_{t}^{k+1}=\frac{{\beta^{\prime }}_{t}}{\sum_{i}{\beta^{\prime }}_{ti}} \end{array}$$

The square brackets in [ln *A*^*T*^ · *s*_*t*_] indicate that this term is used only when observations are available. This speaks to an important aspect of these update schemes; namely, posterior beliefs about the hidden states at all points during the sequence are updated iteratively at each time point. At each time point, the variational updates cycle over representations of future states to update the sufficient statistics encoding posterior beliefs. These update cycles are themselves repeated as time progresses, so that there is convergence both within and between cycles. This means the sufficient statistics change over two timescales; a fast timescale that updates posterior beliefs about the future and a slow timescale that updates posterior beliefs in the future. Posterior beliefs about the trajectory, at both timescales, ensure that the trajectory convergences on the final (desired) location, where the anticipated trajectory is realized through action. It is interesting to speculate about neurophysiologic implementations of this sort of scheme, particularly in relation to nested electrophysiological oscillations (Canolty et al., 2006). The notion here is that the electrophysiological correlates of updating may show nested oscillations, with fast (gamma) oscillations reflecting updates in a fictive future and slower (theta) dynamics that reflect updates in real time; with timescales of 25 and 250 ms respect, respectively. To illustrate the nature of this optimal control, we now apply it to a well-known problem in optimal control theory that presents some special challenges.

### The mountain car problem

In the mountain car problem, one has to park a mountain car halfway up the side of a valley. However, the mountain car is not strong enough to climb directly to the parking place, which means the only way to assess the goal is to ascend the other side of the valley to acquire sufficient momentum during the return trip. This represents an interesting problem, when considered in the state space of position and velocity: the agent has to move away from its target location to attain the goal later. In other words, it has to execute a circuitous trajectory through state space (as in avoiding obstacles). We have used this problem previously to illustrate how Bayes-optimal control can be learned in terms of the parameters controlling prior beliefs about trajectories (Friston et al., 2009) and using heuristic policies (Gigerenzer and Gaissmaier, 2011) based on the destruction of costly fixed point attractors (Friston, 2010).

**Figure 2:**
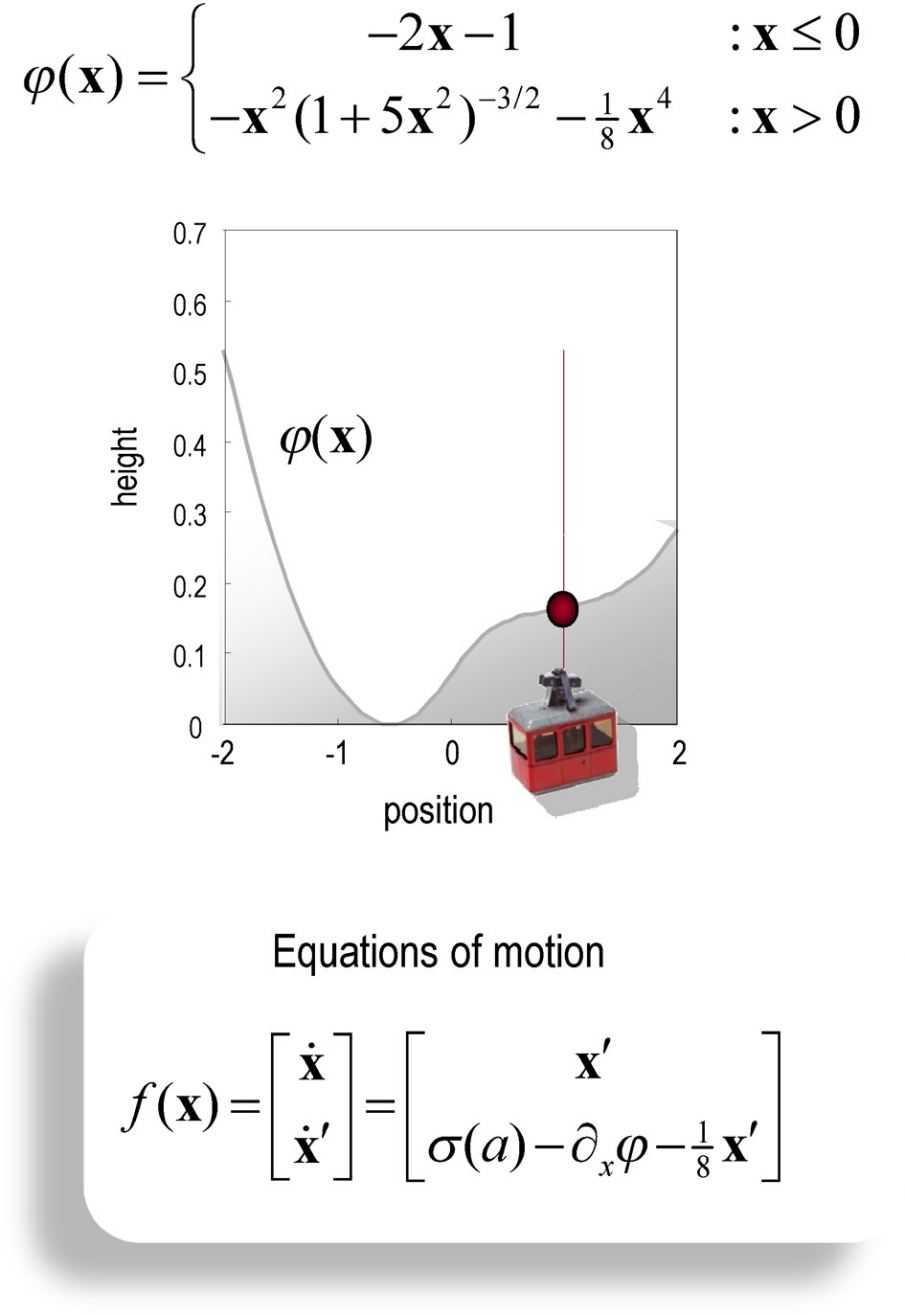
**Schematic of the mountain car problem.** The **upper panel** (and associated equations) illustrate the landscape or potential energy function that defines the motion of the car. This has a minima at *x* = (−0.5, 1). The mountain-car is shown at the desired parking position at the top of the hill on the right *x* = (1, 0) (indicated with a red ball). The equations of motion in the **lower panel** describe the forces exerted on the car, which include σ (*a*), a sigmoid (hyperbolic tangent) function of action, gravitational forces, and friction.

It should be noted that the mountain car problem is normally cast as a learning problem—in which an optimal policy has to be learned. However, here, we use it to illustrate optimal behavior in terms of inference. In other words, we assume the agent has already learned the constraints afforded by the world it operates in—and now has to infer an optimal policy within a single trial. In this setting, the mountain car problem provides a challenging inference problem, particularly when we include random fluctuations in both the states generating observations and the observations themselves. The mountain car problem can be specified with the equations of motion in Figure 2. Here, we consider a discrete state space and time formulation of this problem and use it to illustrate agency based control.

To create a discrete version, we ensured that expected changes in position and velocity match the equations of motion, when integrated over discrete time intervals (here Δ*t* = 2s). The ensuing pullback probabilities for each level of control satisfy (subject to the constraint that only the states adjacent to the expected position and velocity are non-zero).

(14) $$\sum_{i}x(x_{i})B_{ijk}=x({\tilde{x}}_{j})-f(x(x_{j}),a(u_{k}))\Delta t$$

Here, **x**(*x*_*i*_) ∈ ℝ^2^ returns the continuous position and velocity associated with the *i*-th hidden state. Similarly, *a*(*u*_*k*_) ∈ ℝ returns the real valued action associated with the *k*-th control state. In these simulations, we used five levels of control corresponding to *a*(*u*_*k*_) ∈ {−2, −1, 0, 1, 2}. This means the agent assumes that strong or intermediate acceleration can be applied in a right or leftward direction. To simulate random fluctuations in the motion of the mountain car, we smoothed the parameter matrix **B** to augment the uncertainty about the previous state incurred by discretizing state space. The state space comprised 32 position (from −2 to 2) and velocity bins (from −3 to 3), giving 32 × 23 = 1024 discrete states. For simplicity, we assumed a one-to-one mapping between hidden and observed states; that is **A** = *I* and placed uniform prior costs over control. Prior beliefs about the final state specify the goal **x** = (1, 0)—namely, to maintain a position at the parking location with zero velocity; see Figure 2. Finally, the action-dependent sampling probabilities *R*(*s*_*t*+1_|*s*_*t*_, *a*_*t*_) were the transposed versions of the pullback probabilities in Equation (14). These sampling probabilities were used to select action and to generate the next sensory input. Action used the same five levels as the control states—however, as noted above, there is no requirement that action and control be related in this way.

**Figure 3:**
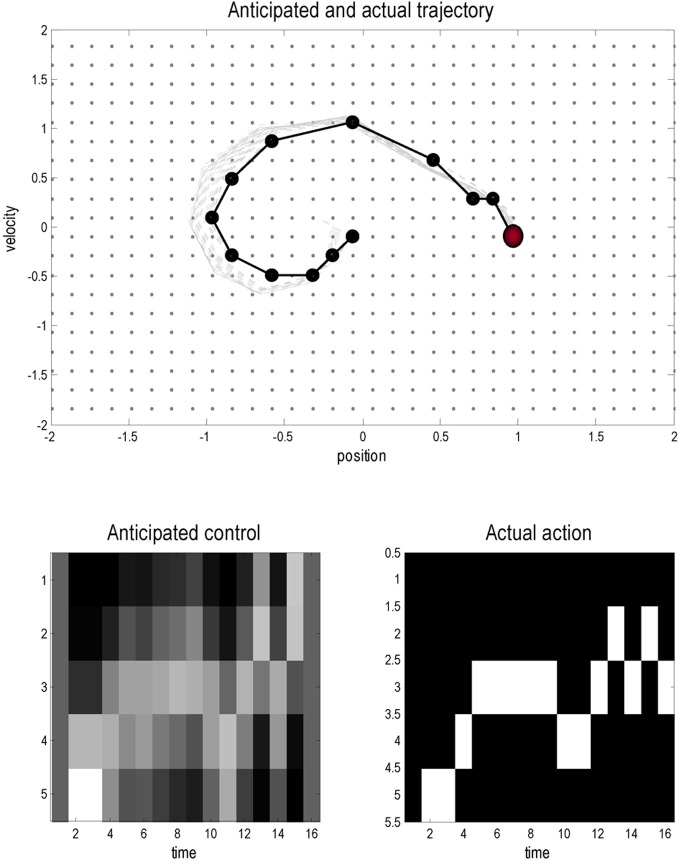
**This figure shows the results of a simulated (agency based) trajectory over *T* = 16 time steps starting at *x* = (0, 0) and ending at the goal location *x* = (1, 0) (red ball) using active inference and explicit representations of the future.** The **upper panel** shows the trajectories in the state space of position and velocity. The grey lines represent anticipated trajectories accumulated during control, while the dark (dotted) lines show the actual trajectory through state space. The anticipated trajectories are the expected values based upon posterior expectations about past and future states. They are therefore continuous functions of position and velocity. In contrast, the actual trajectory is restricted to the 1024 discrete states that can be occupied; these are shown as light grey dots. The **lower panels** show the anticipated control and the actual actions selected under active inference (in image format where lighter colors mean a higher probability). Note that there is a high degree of correspondence; however, the posterior beliefs about control and not always absolutely certain: these are the beliefs at the times each action is selected.

Figure 3 shows the results of a simulation using *T* = 16 time steps and a starting position of **x** = (0, 0). In these simulations the variational updates were repeated eight times and then an action was selected. The upper panel shows the trajectories (real and anticipated) through state space, while the lower panels show the inferred control states and selected action as a function of time. The darker line in the upper panel connects the states visited over the 16 time steps, while the gray lines report the anticipated trajectories from the beginning of the trial to the end. The inferred trajectories are shown as the expected position and velocity, based on posterior beliefs over discrete states. One can see that the actual trajectory fulfills, fairly faithfully, the anticipated sequences and that there has been relatively little updating during execution. As anticipated, the mountain car moves away from its target to acquire sufficient momentum to access the goal on the right. Note the similarity between the selected actions (right) and the inferred control states (left). The interesting thing here is that the agent was not always sure about which control state was currently engaged. However, the control state with the highest posterior probability, which corresponds to the action the agent believes it will emit next, is always selected by active inference. In other words, even under uncertainty about hidden and control states, there is sufficient confidence in the next sensory state to inform action.

### Summary

In summary, we have reviewed conventional approaches to (partially observable) Markov decision problems and have cast reward or cost functions in terms of prior beliefs about state transitions. This implicitly resolves the redundancy between cost functions and priors that underlies the complete class theorems. We then exploited this redundancy by specifying optimal policies in terms of prior beliefs about future (terminal) states. The ensuing scheme may provide a metaphor for model-based decision-making in real agents that has an explicit planning or anticipatory aspect. This solution was based upon approximate (variational) Bayesian inference that respects the Markov nature of decision processes.

The aim of this work was to unpack some of the implications of optimal control for its implementation in real-world agents. The most important is the representation of hidden control states that are required for accessing distal rewards in the future. This contrasts with the usual problem formulation of MDPs, which is to define a normative model and a corresponding notion of optimality. In optimal control theory, state transitions are specified in terms of value functions that are solutions to the appropriate Bellman optimality equations, given a cost function. The notion that the Bellman optimality principle “can be derived as a limit case” from the variational principles that underlie active inference also emerges in recent information theoretic formulations of bounded rationality (Braun et al., 2011): Braun et al. consider control costs in terms of the (cross) entropy of choice probabilities and augment expected utility to produce a free energy optimality criterion. This *free utility* captures bounded rationality by ensuring the divergence between optimal and prior choice probabilities is minimized. They show that minimizing free utility includes both discrete and continuous stochastic optimal control as special cases and can be derived “without invoking the Hamilton–Jacobi–Bellman equation or the Bellman optimality equations”. See also Theodorou et al. (2010), who exploit a similar formalism but with a more classical motivation. The generalization of optimal control using free utility is compelling and unifies approximate optimal control methods in both the continuous and discrete domain. However, free utility is fundamentally different from variational free energy, because it is a functional of choice probabilities over hidden states. In contrast, variational free energy is a function of observed states. Crucially, free utility depends on a cost function, while free energy does not. This is because the free energy principle is based on the invariant or ergodic solution *P*(*s*|*m*) to the *Kolmogorov forward equation*, which specifies the value of an observed state *V*(*s*|*m*) = ln *P*(*s*|*m*) directly, without reference to cost—see next section and Friston and Ao (2012). In other words, value is (log) evidence or negative surprise. Conversely, free utility is based on the *Kolmogorov backward equation*, which can only be solved given terminal costs.

In answer to the title of this paper, the value of an observed state is then prescribed by a generative model in terms of the probability a state will be occupied. It can be seen easily that minimizing the entropy of the invariant probability distribution over observations maximizes expected value:

(15) $$E_{P}[-\ln P(s|m)]=E_{P}[V(s|m)]$$

Minimizing the entropy of observed states is the *raison d'être* for the free energy principle (see below), which invokes variational free energy to finesse the intractable problem of marginalizing over hidden states to evaluate value or negative surprise. This complements the use of free utility to finesse the intractable problem of solving Bellman optimality equations (Braun et al., 2011). It can be seen from Equation (5) that free energy *F*(*s*, μ) ≥ −ln *P*(*s*|*m*) = −*V*(*s*|*m*) bounds surprise and can therefore be minimized to maximize value.

In conclusion, we have described a variational free energy formulation of (partially observable) Markov decision problems in decision making under uncertainty. We have seen that optimal control can be cast as *active inference*, in which both *action and posterior beliefs* about hidden states minimize a free energy bound on the value (log Bayesian model evidence) of observed states, under a generative model. In this setting, reward or cost functions are absorbed into prior beliefs about state transitions and terminal states. This converts optimal control into a pure inference problem, enabling the application of standard Bayesian filtering techniques. Crucially, this entails modeling future states state that endows the generative model with a sense of agency. This leads to a distinction between models with and without inference on future states—namely, agency free and agency based models, respectively. In the next section, we ask: where do prior beliefs about future states come from?

## Action, perception, and control

The previous section suggested that value is simply the log-evidence associated with sensory samples or evidence for an internal model or hypothesis about the world. In this setting, valuable behavior simply involves sampling the world to ensure model predictions are fulfilled, where these predictions rest upon (prior) beliefs about future states. In this section, we motivate the imperative to maximize log-evidence from the basic principles of self-organization. We go on to show that prior beliefs about future states have a relatively simple form; namely, we believe that our future states will minimize uncertainty about our current beliefs.

If perception corresponds to hypothesis testing (Gregory, 1980); then sensory sampling might be correspond to experiments that generate sensory data. In the next three sections, we explore the idea that eye movements are optimal experiments, in which data are gathered to test hypotheses or beliefs about how those data are caused. This provides a plausible model of visual search that can be motivated from the basic tenets of self-organized behavior: namely, the imperative to minimize the entropy of hidden states of the world and their sensory consequences. Simulations of the resulting active inference scheme reproduce sequential eye movements that are reminiscent of empirically observed saccades and provide some counterintuitive insights into the way that sensory evidence is accumulated or assimilated into beliefs about the world.

If variational free energy minimization is applied to both action and perception, action will fulfill predictions based upon conditional beliefs about the state of the world. However, the uncertainty associated with those conditional beliefs depends upon the way data are sampled: for example, where we direct our gaze or how we palpate a surface. The deployment of sensory epithelia is itself a hidden state that has to be inferred. However, these hidden states can be changed by action, which means there is a subset of hidden states over which we have control. These are the hidden control states of the previous section. Prior beliefs about these hidden control states dictate how we engage actively with the environment and lead to the notion of fictive or *counterfactual representations*; in other words, what we would infer about the world, if we sampled it in a particularly way. This leads naturally to the internal representation of prior beliefs about future sampling and the emergence of things like agency, intention, and salience. We will illustrate these points using visual search and the optimal control of saccadic eye movements (Grossberg et al., 1997; Itti and Baldi, 2009; Srihasam et al., 2009); noting that similar principles should apply to other sensory modalities. For example, they should apply to motor control when making inferences about objects causing somatosensory sensations (Gibson, 1979).

### Active inference—a continuous time formulation

This section establishes the nature of Bayes-optimal inference in the context of controlled sensory searches. It starts with the basic premise that underlies free energy minimization; namely, the imperative to minimize the dispersion of sensory states and their hidden causes to ensure a homoeostasis of the external and internal milieu (Ashby, 1947). It rehearses briefly how action and perception follow from this imperative and highlights the important role of prior beliefs about the sampling of sensory states. At this point, we move away from the discrete formulations of MDPs and turned to continuous formulations, where probability distributions become densities and discrete time becomes continuous. This shift is deliberate and allows the discrete formulations of the previous sections to be compared and contrasted with the equivalent continuous time formulations that predominate in biologically realistic simulations.

**Notation and set up:** Here we use *X*:Ω × … → ℝ for real valued random variables and *x* ∈ *X* for particular values. A probability density will be denoted by *p*(*x*) = Pr{*X* = *x*} using the usual conventions and its entropy *H*[*p*(*x*)] by *H*(*X*). From now on, the tilde notation $\tilde{x}=(x,\;x^{\prime },\;x^{\prime \prime },\;\ldots )$ denotes variables in generalized coordinates of motion (Friston, 2008), where each prime denotes a temporal derivative (using Lagrange's notation). For simplicity, constant terms will be omitted from equalities.

**Definition:** Active inference rests on the tuple (Ω, Ψ, *S*, *A*, *R*, *q*, *p*) that comprises the following:

- *A sample space* Ω or non-empty set from which random fluctuations or outcomes ω ∈ Ω are drawn
- *Hidden states* Ψ:Ψ × *A* × Ω → ℝ—states of the world that cause sensory states and depend on action
- *Sensory states S*:Ψ × *A* × Ω → ℝ—the agent's sensations that constitute a probabilistic mapping from action and hidden states
- *Action A*:*S* × *R* → ℝ—an agent's action that depends on its sensory and internal states
- *Internal states R*:*R* × *S* × Ω → ℝ—the states of the agent that cause action and depend on sensory states
- *Generative density* $p(\tilde{s},\;\tilde{\psi }|m)$—a probability density function over sensory and hidden states under a generative model denoted by *m*
- *Conditional density* $q(\tilde{\psi }):=q(\tilde{\psi }|\tilde{\mu })$—an arbitrary probability density function over hidden states $\tilde{\psi }\in \Psi$ that is parameterized by internal states $\tilde{\mu }\in R$

We assume that the imperative for any biological system is to minimize the dispersion of its sensory and hidden states, with respect to action (Ashby, 1947; Nicolis and Prigogine, 1977; Friston and Ao, 2012). We will refer to the sensory and hidden states collectively as *external states S* × Ψ. As noted above, the dispersion of external states corresponds to the (Shannon) entropy of their probability density that, under ergodic assumptions, equals (almost surely) the long-term time average of a Gibbs energy:

(16) $$\begin{array}{l} H(S,\Psi )=E_{t}[G(\tilde{s}(t),\tilde{\psi }(t))]\\ \;G=-\ln p(\tilde{s}(t),\tilde{\psi }(t)|m) \end{array}$$

Gibbs energy $G(\tilde{s},\;\tilde{\psi })$ is defined in terms of the generative density or model. Clearly, agents cannot minimize this energy directly because the hidden states are unknown. However, we can decompose the entropy into the entropy of the sensory states (to which the system has access) and the conditional entropy of hidden states (to which the system does not have access). This second term is also called the *equivocation* of the hidden states about the sensory states:

(17) $$\begin{array}{l} H(S,\Psi )=H(S)+H(\Psi |S)\\ \;=E_{t}[-\ln p(\tilde{s}(t)|m)+H(\Psi |S=\tilde{s}(t))] \end{array}$$

This decomposition means that the entropy of the external states can be minimized through action to minimize sensory surprise $-\ln p(\tilde{s}(t)|m)$, under the assumption that the consequences of action minimize the equivocation or average uncertainty about hidden states:

(18) $$\begin{array}{l} a(t)=\underset{a\in A}{\arg\min}\{-\ln p(\tilde{s}(t)|m)\}\\ \tilde{u}(t)=\underset{\tilde{u}\in U}{\arg\min}\{H(\Psi |S=\tilde{s}(t))\} \end{array}$$

The consequences of action are expressed by changes in a subset of hidden states *U* ⊂ Ψ—the hidden control states or *hidden controls.* When Equation (18) is satisfied, the variation of entropy in Equation (16) with respect to action and its consequences are zero, which means the entropy has been minimized (at least locally). However, the hidden controls cannot be optimized explicitly because they are hidden from the agent. To resolve this problem, we first consider action and then return to optimizing hidden control states.

### Action and perception

Action cannot minimize sensory surprise directly because this would involve an intractable marginalization over hidden states, so—as in the discrete formulation—surprise is replaced with an upper bound called variational free energy (Feynman, 1972). However, replacing surprise with free energy means that internal states also have to minimize free energy, because free energy is a function of internal states:

(19) $$\begin{array}{l} a(t)=\underset{a\in A}{\arg\min}\{F(\tilde{s}(t),\tilde{\mu }(t))\}\\ \tilde{\mu }(t)=\underset{\tilde{\mu }\in R}{\arg\min}\{F(\tilde{s}(t),\tilde{\mu })\}\\ \;F=E_{q}[G(\tilde{s},\tilde{\psi })]-H[q(\tilde{\psi }|\tilde{\mu })]\\ \;=-\ln p(\tilde{s}|m)+D[q(\tilde{\psi })||p(\tilde{\psi }|\tilde{s},m)]\\ \;\geq -\ln p(\tilde{s}|m) \end{array}$$

This induces a dual minimization with respect to action and the internal states that parameterize the conditional density. These minimizations correspond to action and perception, respectively. In brief, the need for perception is induced by introducing free energy to finesse the evaluation of surprise; where free energy can be evaluated by an agent fairly easily, given a generative model. The last equality says that free energy is always greater than surprise because the second (Kullback–Leibler divergence) term is non-negative. As in the discrete formulation, when free energy is minimized with respect to the internal states, free energy approximates surprise and the conditional density approximates the posterior density over external states:

(20) $$D[q(\tilde{\psi })||p(\tilde{\psi }|\tilde{s},m)]\approx 0\Rightarrow \{\begin{array}{c} q(\tilde{\psi })\approx p(\tilde{\psi }|\tilde{s},m)\\ H[q(\tilde{\psi })]\approx H(\Psi |S=\tilde{s}) \end{array}$$

Minimizing free energy also means that the entropy of the conditional density approximates the equivocation of the hidden states. This allows us to revisit the optimization of hidden controls, provided we know how they affect the conditional density.

### The maximum entropy principle and the Laplace assumption

If we admit an encoding of the conditional density up to second order moments, then the maximum entropy principle (Jaynes, 1957) implicit in the definition of free energy (Equation 19) requires $q(\tilde{\psi }|\tilde{\mu })=N(\tilde{\mu },\;\Sigma )$ to be Gaussian. This is because a Gaussian density has the maximum entropy of all forms that can be specified with two moments. Adopting a Gaussian form is known as the Laplace assumption and enables us to express the entropy of the conditional density in terms of its first moment or expectation. This follows because we can minimize free energy with respect to the conditional covariance as follows:

(21) $$\begin{array}{l} \;F=G(\tilde{s},\tilde{\mu })+\frac{1}{2}tr(\Sigma \cdot \partial_{\tilde{\mu }\tilde{\mu }}G)-\frac{1}{2}\ln|\Sigma |\\ \;\Rightarrow \;\partial_{\Sigma }F=\frac{1}{2}\partial_{\tilde{\mu }\tilde{\mu }}G-\frac{1}{2}\Pi \\ \partial_{\Sigma }F=0\Rightarrow \Pi =\partial_{\tilde{\mu }\tilde{\mu }}G\Rightarrow H(\Psi |S=\tilde{s})\\ \;\approx H[q(\tilde{\psi })]=-\frac{1}{2}\ln|\partial_{\tilde{\mu }\tilde{\mu }}G| \end{array}$$

Here, the conditional precision $\Pi (\tilde{s},\tilde{\mu })$ is the inverse of the conditional covariance $\Sigma (\tilde{s},\tilde{\mu })$. In short, the entropy of the conditional density and free energy are functions of the conditional expectations and sensory states. Now that we have (an approximation to) the equivocation, we can return to its minimization through prior beliefs.

### Bayes-optimal control

We can now optimize the hidden controls vicariously through prior expectations that are fulfilled by action. This can be expressed in terms of prior expectations about hidden controls.

(22) $${\tilde{\eta }}_{u}(t)=\underset{{\tilde{\eta }}_{u}\in U}{\arg\min}\{H[q(\tilde{\psi }|{\tilde{\mu }}_{x}(t+\tau ),{\tilde{\eta }}_{u})]\}$$

This equation means the agent expects hidden control states to minimize uncertainty about hidden states in the future—this is the entropy of the conditional density in the future, which we will call a counterfactual density. Interestingly, Equations (19) and (22) say that conditional expectations (about hidden states) maximize conditional uncertainty, while prior expectations (about hidden controls) minimize conditional uncertainty. This means the posterior and prior beliefs are in opposition, trying to maximize and minimize uncertainty about hidden states, respectively. The latter represent prior beliefs that hidden states are sampled to maximize conditional confidence, while the former minimizes conditional confidence to ensure the explanation for sensory data does not depend on particular hidden states—in accord with the maximum entropy principle (or Laplace's principle of indifference). In what follows, we will refer to the negative entropy of the counterfactual density as *salience*; noting that salience is a measure of confidence about hidden states that depends on how they are sampled. This means that the agent believes, a priori, that salient features will be sampled.

### Summary and related principles

To recap, we started with the assumption that biological systems minimize the dispersion or entropy of states in their external milieu to ensure a sustainable and homoeostatic exchange with their environment (Ashby, 1947). Clearly, these states are hidden and therefore cannot be measured or changed directly. However, if agents know how their action changes sensations (for example, if they know contracting certain muscles will necessarily excite primary sensory afferents from stretch receptors), then they can minimize the dispersion of their sensory states by countering surprising deviations from expected values. However, reducing the dispersion of sensory states will only reduce the dispersion of hidden states, if the sensory states report the underlying hidden states faithfully. This faithful reporting requires agents to minimize their conditional uncertainty about hidden states, through prior beliefs about the way sensory organs are deployed. This imperative—to minimize conditional uncertainty—is remarkably consistent with a number of other constructs, such as Bayesian surprise (Itti and Baldi, 2009). It is fairly easy to show that maximizing salience is the same as maximizing Bayesian surprise (Friston et al., 2012a). This is important because it links salience in the context of active inference with salience in the theoretical (Humphreys et al., 2009) and empirical literature (Shen et al., 2011; Wardak et al., 2011). Here, we will focus on the principle of maximum mutual information.

Priors about hidden controls express the belief that conditional uncertainty will be minimal. The long-term average of this conditional uncertainty is the conditional entropy of hidden states, which can be expressed as the entropy over hidden states minus the mutual information between hidden and sensory states:

(23) $$H(\Psi |S)=E_{t}[H(\Psi |S=\tilde{s}(t))]=H(\Psi )-I(\Psi ;S)$$

In other words, minimizing conditional uncertainty is equivalent to maximizing the mutual information between external states and their sensory consequences. This is one instance of the Infomax principle (Linsker, 1990). Previously, we have considered the relationship between free energy minimization and the principle of maximum mutual information, or minimum redundancy (Barlow, 1961, 1974; Optican and Richmond, 1987; Oja, 1989; Olshausen and Field, 1996; Bialek et al., 2001) in terms of the mapping between hidden and internal states (Friston, 2010). In this setting, one can show that “the infomax principle is a special case of the free-energy principle that obtains when we discount uncertainty and represent sensory data with point estimates of their causes.” Here, we consider the mapping between external and sensory states and find that prior beliefs about how sensory states are sampled further endorse the Infomax principle. In what follows, we consider the neurobiological implementation of these principles.

## Neurobiological implementations of active inference

In this section, we take the general principles above and consider how they might be implemented in a (simulated) brain. The equations in this section may appear a bit complicated; however, they are based on just four assumptions.

- The brain minimizes the free energy of sensory inputs defined by a generative model.
- This model includes prior expectations about hidden controls that maximize salience.
- The generative model used by the brain is hierarchical, non-linear, and dynamic.
- Neuronal firing rates encode the expected state of the world, under this model.

The first assumption is the free energy principle, which leads to active inference in the embodied context of action. The second assumption follows from the arguments of the previous section. The third assumption is motivated easily by noting that the world is both dynamic and non-linear and that hierarchical causal structure emerges inevitably from a separation of temporal scales (Ginzburg and Landau, 1950; Haken, 1983). Finally, the fourth assumption is the Laplace assumption that, in terms of neural codes, leads to the *Laplace code* that is arguably the simplest and most flexible of all neural codes (Friston, 2009).

Given these assumptions, one can simulate a whole variety of neuronal processes by specifying the particular equations that constitute the brain's generative model. The resulting perception and action are specified completely by the above assumptions and can be implemented in a biologically plausible way as described below (see Table 1 for a list of previous applications of this scheme). In brief, these simulations use differential equations that minimize the free energy of sensory input using a generalized gradient descent (Friston et al., 2010a).

(24) $$\begin{array}{l} \dot{\tilde{\mu }}(t)=D\tilde{\mu }(t)-\partial_{\tilde{\mu }}F(\tilde{s},\tilde{\mu })\\ \dot{a}(t)=-\partial_{a}F(\tilde{s},\tilde{\mu }) \end{array}$$

**Table 1:** **Processes and paradigms that have been modeled using the generalized Bayesian filtering scheme in this paper**.

**Domain**	**Process or paradigm**
Perception	Perceptual categorization (bird songs) (Friston and Kiebel, 2009a,b)
	Novelty and omission-related responses (Friston and Kiebel, 2009a,b)
	Perceptual inference (speech) (Kiebel et al., 2009)
Sensory learning	Perceptual learning (mismatch negativity) (Friston and Kiebel, 2009a,b)
Attention	Attention and the Posner paradigm (Feldman and Friston, 2010)
	Attention and biased competition (Feldman and Friston, 2010)
Motor control	Retinal stabilization and oculomotor reflexes (Friston et al., 2010b)
	Saccadic eye movements and cued reaching (Friston et al., 2010b)
	Motor trajectories and place cells (Friston et al., 2011)
Sensorimotor integration	Bayes-optimal sensorimotor integration (Friston et al., 2010b)
Behavior	Heuristics and dynamical systems theory (Friston and Ao, 2012)
	Goal-directed behavior (Friston et al., 2009)
Action observation	Action observation and mirror neurons (Friston et al., 2011)

These coupled differential equations describe perception and action, respectively, and just say that internal brain states and action change in the direction that reduces free energy. The first is known as generalized predictive coding and has the same form as Bayesian (e.g., Kalman–Bucy) filters used in time series analysis; see also Rao and Ballard (1999). The first term in Equation (24) is a prediction based upon a differential matrix operator D that returns the generalized motion of the expectation, such that $D\tilde{\mu }={\left[\mu^{\prime },\;\mu^{\prime \prime },\;\mu^{\prime \prime \prime },\;\ldots \right]}^{T}$. The second term is usually expressed as a mixture of prediction errors that ensures the changes in conditional expectations are Bayes-optimal predictions about hidden states of the world. The second differential equation says that action also minimizes free energy. The differential equations above are coupled because sensory input depends upon action, which depends upon perception through the conditional expectations. This circular dependency leads to a sampling of sensory input that is both predicted and predictable, thereby minimizing free energy and surprise.

To perform neuronal simulations under this scheme, it is only necessary to integrate or solve Equation (24) to simulate the neuronal dynamics that encode conditional expectations and ensuing action. Conditional expectations depend upon the brain's generative model of the world, which we assume has the following hierarchical form.

(25) $$\begin{array}{l} \;s=g^{\left(1\right)}(x^{\left(1\right)},v^{\left(1\right)},u^{\left(i\right)})+\omega_{v}^{\left(1\right)}\\ \;{\dot{x}}^{\left(1\right)}=f^{\left(1\right)}(x^{\left(1\right)},v^{\left(1\right)},u^{\left(i\right)})+\omega_{x}^{\left(1\right)}\\ \;\vdots \\ v^{\left(i-1\right)}=g^{\left(i\right)}(x^{\left(i\right)},v^{\left(i\right)},u^{\left(i\right)})+\omega_{v}^{\left(i\right)}\\ \;{\dot{x}}^{\left(i\right)}=f^{\left(i\right)}(x^{\left(i\right)},v^{\left(i\right)},u^{\left(i\right)})+\omega_{x}^{\left(i\right)}\\ \;\vdots \end{array}$$

This equation is just a way of writing down a model that specifies a probability density over the sensory and hidden states, where the hidden states Ψ = *X* × *V* × *U* have been divided into hidden dynamic, causal, and control states. Here, (*g*^(*i*)^, *f*^(*i*)^) are non-linear functions of hidden states that generate sensory inputs at the first level. Hidden causes *V* ⊂ Ψ can be regarded as functions of hidden dynamic states; hereafter, hidden states *X* ⊂ Ψ. Random fluctuations (ω^(*i*)^_*x*_, ω^(*i*)^_*v*_) on the motion of hidden states and causes are conditionally independent and enter each level of the hierarchy. It is these that make the model probabilistic: they play the role of sensory noise at the first level and induce uncertainty about states at higher levels. The inverse amplitudes of these random fluctuations are quantified by their precisions (Π^(*i*)^_*x*_, Π^(*i*)^_*v*_). Hidden causes link hierarchical levels, whereas hidden states link dynamics over time. Hidden states and causes are abstract quantities (like the motion of an object in the field of view) that the brain uses to explain or predict sensations. In hierarchical models of this sort, the output of one level acts as an input to the next. This input can produce complicated (generalized) convolutions with deep (hierarchical) structure.

### Perception and predictive coding

Given the form of the generative model (Equation 25) we can now write down the differential equations (Equation 24) describing neuronal dynamics in terms of (precision-weighted) prediction errors on the hidden causes and states. These errors represent the difference between conditional expectations and predicted values, under the generative model (using *A* · *B*: = *A*^*T*^ *B* and omitting higher-order terms):

(26) $$\begin{array}{c} {\dot{\tilde{\mu }}}_{x}^{\left(i\right)}=D{\tilde{\mu }}_{x}^{\left(i\right)}+\frac{\partial {\tilde{g}}^{\left(i\right)}}{\partial {\tilde{\mu }}_{x}^{\left(i\right)}}\cdot \xi_{v}^{\left(i\right)}+\frac{\partial {\tilde{f}}^{\left(i\right)}}{\partial {\tilde{\mu }}_{x}^{\left(i\right)}}\cdot \xi_{x}^{\left(i\right)}-D^{T}\xi_{x}^{\left(i\right)}\;\\ \begin{array}{c} {\dot{\tilde{\mu }}}_{v}^{\left(i\right)}=D{\tilde{\mu }}_{v}^{\left(i\right)}+\frac{\partial {\tilde{g}}^{\left(i\right)}}{\partial {\tilde{\mu }}_{v}^{\left(i\right)}}\cdot \xi_{v}^{\left(i\right)}+{\frac{\partial {\tilde{f}}^{\left(i\right)}}{\partial {\tilde{\mu }}_{v}^{\left(i\right)}}}^{\text{​​}T}\cdot \xi_{x}^{\left(i\right)}-\xi_{v}^{\left(i+1\right)}\\ \begin{array}{l} {\dot{\tilde{\mu }}}_{u}^{\left(i\right)}=D{\tilde{\mu }}_{u}^{\left(i\right)}+\frac{\partial {\tilde{g}}^{\left(i\right)}}{\partial {\tilde{\mu }}_{u}^{\left(i\right)}}\cdot \xi_{v}^{\left(i\right)}+\frac{\partial {\tilde{f}}^{\left(i\right)}}{\partial {\tilde{\mu }}_{u}^{\left(i\right)}}\cdot \xi_{x}^{\left(i\right)}-\xi_{u}^{\left(i+1\right)}\\ \xi_{x}^{\left(i\right)}=\Pi_{x}^{\left(i\right)}(D{\tilde{\mu }}_{x}^{\left(i\right)}-{\tilde{f}}^{\left(i\right)}({\tilde{\mu }}_{x}^{\left(i\right)},{\tilde{\mu }}_{v}^{\left(i\right)},{\tilde{\mu }}_{u}^{\left(i\right)}))\\ \xi_{v}^{\left(i\right)}=\Pi_{v}^{\left(i\right)}({\tilde{\mu }}_{v}^{\left(i-1\right)}-{\tilde{g}}^{\left(i\right)}({\tilde{\mu }}_{x}^{\left(i\right)},{\tilde{\mu }}_{v}^{\left(i\right)},{\tilde{\mu }}_{u}^{\left(i\right)}))\\ \xi_{u}^{\left(i\right)}=\Pi_{u}^{\left(i\right)}({\tilde{\mu }}_{u}^{\left(i-1\right)}-{\tilde{\eta }}_{u}^{\left(i\right)}) \end{array} \end{array} \end{array}$$

Equation (26) can be derived fairly easily by computing the free energy for the hierarchical model in Equation (25) and inserting its gradients into Equation (24). This produces a relatively simple update scheme, in which conditional expectations are driven by a mixture of prediction errors, where prediction errors are defined by the equations of the generative model.

**Figure 4:**
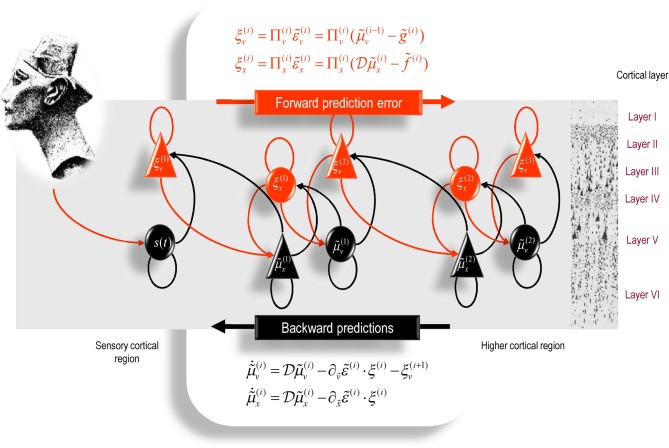
**Schematic detailing the neuronal architecture that might encode conditional expectations about the states of a hierarchical model.** This shows the speculative cells of origin of forward driving connections that convey prediction error from a lower area to a higher area and the backward connections that construct predictions (Mumford, 1992). These predictions try to explain away prediction error in lower levels. In this scheme, the sources of forward and backward connections are superficial and deep pyramidal cells, respectively. The equations represent a generalized descent on free-energy under the hierarchical models described in the main text: see also Friston (2008). State-units are in black and error-units in red. Here, neuronal populations are deployed hierarchically within three cortical areas (or macro-columns). Within each area, the cells are shown in relation to cortical layers: supra-granular (I–III), granular (IV), and infra-granular (V and VI) layers. For simplicity, conditional expectations about control states had been absorbed into conditional expectations about hidden causes.

It is difficult to overstate the generality and importance of Equation (26): its solutions grandfather nearly every known statistical estimation scheme, under parametric assumptions about additive or multiplicative noise (Friston, 2008). These range from ordinary least squares to advanced variational deconvolution schemes. The resulting scheme is called *generalized filtering* or predictive coding (Friston et al., 2010a). In neural network terms, Equation (26) says that error-units receive predictions from the same level and the level above. Conversely, conditional expectations (encoded by the activity of state units) are driven by prediction errors from the same level and the level below. These constitute bottom–up and lateral messages that drive conditional expectations toward a better prediction to reduce the prediction error in the level below. This is the essence of recurrent message passing between hierarchical levels to optimize free energy or suppress prediction error: see Friston and Kiebel (2009a) for a more detailed discussion. In neurobiological implementations of this scheme, the sources of bottom–up prediction errors are thought to be superficial pyramidal cells that send forward connections to higher cortical areas. Conversely, predictions are conveyed from deep pyramidal cells, by backward connections, to target (polysynaptically) the superficial pyramidal cells encoding prediction error (Mumford, 1992; Friston and Kiebel, 2009a). Figure 4 provides a schematic of the proposed message passing among hierarchically deployed cortical areas.

### Action

In active inference, conditional expectations elicit behavior by sending top–down predictions down the hierarchy that are unpacked into proprioceptive predictions at the level of the cranial nerve nuclei and spinal-cord. These engage classical reflex arcs to suppress proprioceptive prediction errors and produce the predicted motor trajectory.

(27) $$\dot{a}=-\frac{\partial }{\partial a}F=-\frac{\partial \tilde{s}}{\partial a}\cdot \xi_{v}^{\left(1\right)}$$

The reduction of action to classical reflexes follows because the only way that action can minimize free energy is to change sensory (proprioceptive) prediction errors by changing sensory signals; cf., the equilibrium point formulation of motor control (Feldman and Levin, 1995). In short, active inference can be regarded as equipping a generalized predictive coding scheme with classical reflex arcs: see Friston et al. (2009, 2010b) for details. The actual movements produced clearly depend upon top–down predictions that can have a rich and complex structure.

### Counterfactual processing

To optimize prior expectations about hidden controls it is necessary to identify those that maximize the salience. We will focus on visual searches and assume that competing (counterfactual) prior expectations are represented explicitly in a saliency map. In other words, we assume that salience is encoded on a grid corresponding to discrete values of competing prior expectations associated with different hidden control states. The maximum of this map defines the prior expectation with the greatest salience. This prior expectation enters the predictive coding in Equation (25). The salience of the *j*-th counterfactual prior expectation is, from Equations (21) and (22),

(28) $$\begin{array}{l} {\tilde{\eta }}_{u}(t)=\arg\max_{{\tilde{\eta }}_{j}}S({\tilde{\eta }}_{j})\\ S({\tilde{\eta }}_{j})=\frac{1}{2}\ln|\partial_{\tilde{\mu }\tilde{\mu }}G({\tilde{\mu }}_{x}(t+\tau ),{\tilde{\mu }}_{v}(t+\tau ),{\tilde{\eta }}_{j})| \end{array}$$

Given that we will be simulating visual searches with saccadic eye movements, we will consider the prior expectations to be updated at discrete times to simulate successive saccades, where the hidden control states correspond to locations in the visual scene that attract visual fixation.

### Summary

In summary, we have derived equations for the dynamics of perception and action using a free energy formulation of adaptive (Bayes-optimal) exchanges with the world and a generative model that is generic and biologically plausible. In what follows, we use Equations (26), (27), and (28) to simulate neuronal and behavioral responses. A technical treatment of the material above can be found in Friston et al. (2010a), which provides the details of the generalized Bayesian filtering scheme used to produce the simulations in the next section. The only addition to previous illustrations of this scheme is Equation (28), which maps conditional expectations about hidden states to prior expectations about hidden controls: it is this mapping that underwrites the sampling of salient features and appeals to the existence of hidden control states that action can change. Put simply, this formulation says that action fulfills predictions and we predict that the consequences of action (hidden control states) minimize our uncertainty about predictions.

## Modeling saccadic eye movements

This section illustrates the theory of the previous section, using simulations of sequential eye movements. Saccadic eye movements are a useful vehicle to illustrate active inference because they speak directly to visual search strategies and a wealth of psychophysical, neurobiological, and theoretical study (e.g., Grossberg et al., 1997; Ferreira et al., 2008; Srihasam et al., 2009; Bisley and Goldberg, 2010; Shires et al., 2010; Tatler et al., 2011; Wurtz et al., 2011). We will focus on a fairly simple paradigm—the categorization of faces—and therefore sidestep many of the deeper challenges of understanding visual searches.

### The generative process

That first thing that we need to do is to define the processes generating sensory signals as a function of (hidden) states and action:

(29) $$\begin{array}{l} s_{p}=x_{p}+\omega_{v,p}\\ s_{q}=g(I,x_{p})+\omega_{v,q}\\ g_{i}=I(d_{i,1}+x_{p,1},d_{i,2}+x_{p,2})\cdot h_{i}\\ {\dot{x}}_{p}=a-\frac{1}{16}x_{p}+\omega_{x,p} \end{array}$$

Note that these hidden states are true states that actually produce sensory signals. These have been written in boldface to distinguish them from the hidden states assumed by the generative model (see below). In these simulations, the world is actually very simple: sensory signals are generated in two modalities—proprioception and vision. Proprioception, *s*_*p*_ ∈ ℝ^2^ reports the center of gaze or foveation as a displacement from the origin of some extrinsic frame of reference. Inputs in the visual modality comprise a list *s*_*q*_ ∈ ℝ^256^ of values over an array of sensory channels sampling a two-dimensional image or visual scene *I* : ℝ^2^ → ℝ. This sampling uses a grid of 16 × 16 channels that samples a small part the image—representing a local high-resolution (foveal) sampling that constitutes an attentional focus. To make this sampling more biologically realistic, each channel was equipped with a center-surround receptive field that samples a local weighted average of the image. This provides an on-off center-surround sampling. Furthermore, the signals are modulated by a two-dimensional Hamming function—to model the loss of precise visual information from the periphery of the visual field.

The only hidden states in this generative process **x**_*p*_ ∈ ℝ^2^ are the center of oculomotor fixation, whose motion is driven by action and decays with a suitably long time constant of 16 time bins (were a time bin corresponds to 12 ms). In practice, the visual scene corresponds to a large grayscale image, where the *i*-th visual channel is sampled at location *d*_*i*_ + **x**_*p*_ ∈ ℝ^2^. Here, *d*_*i*_ ∈ ℝ^2^ specifies the displacement of the *i*-th channel from the center of the sampling grid. The proprioceptive and visual signals were effectively noiseless, where there random fluctuations had a log-precision of 16. The motion of the fixation point was subject to low amplitude fluctuations with a log-precision of eight. This completes our description of the process generating proprioceptive and visual signals for any given action. We now turn to the model of this process that generates predictions and action.

### The generative model

The model of sensory signals used to specify variational free energy and consequent action (visual sampling) is slightly more complicated than the actual process generating data:

(30) $$\begin{array}{l} s_{p}=x_{p}+\omega_{v,p}\\ s_{q}=\sum_{i}\exp(x_{q,i})g(I_{i},x_{p})+\omega_{v,q}\\ {\dot{x}}_{p}=\frac{1}{4}(u-x_{p})+\omega_{x,p}\\ {\dot{x}}_{q}=1-\sum_{i}\exp(x_{q,i})-\frac{1}{1024}x_{q}+\omega_{x,p} \end{array}$$

As above, proprioceptive signals are just a noisy mapping from hidden proprioceptive states encoding the direction of gaze. The visual input is modeled as a mixture of images sampled at a location specified by the proprioceptive hidden state. This hidden state decays with a time constant of four time bins (48 ms) toward a hidden control state. In other words, the hidden control determines the location that attracts gaze.

The visual input depends on a number of hypotheses or internal images *I*_*i*_ : ℝ^2^ → ℝ : *i* ∈ {1, … *N*} that constitute the agent's prior beliefs about what could cause its visual input. In this paper, we use *N* = 3 hypotheses. The input encountered at any particular time is a weighted mixture of these internal images, where the weights correspond to hidden perceptual states. The dynamics of these perceptual states (last equality above) implement a form of dynamic softmax—in the sense that the solution of their equations of motion ensures the weights sum (approximately) to one:

(31) $${\dot{x}}_{q}=0\Rightarrow \sum_{i}\exp(x_{q,i})\approx 1$$

This means we can interpret exp(*x*_*q*, *i*_) as the (softmax) probability that the *i*-th internal image or hypothesis is the cause of visual input. The decay term (with a time constant of 512 time bins) just ensures that perceptual states decay slowly to the same value, in the absence of perceptual fluctuations.

In summary, given hidden proprioceptive and perceptual states the agent can predict its proprioceptive and visual input. The generative model is specified by Equation (17) and the precision of the random fluctuations that determine the agent's prior certainty about sensory inputs and the motion of hidden states. In the examples below, we used a log-precision of eight for proprioceptive sensations and the motion of hidden states. We let the agent believe its visual input was fairly noisy, with a log-precision of four. In practice, this means it is more likely to change its (less precise) posterior beliefs about the causes of visual input to reduce prediction error, as opposing to adjusting its (precise) posterior beliefs about where it is looking.

### Priors and saliency

To simulate saccadic eye movements, we integrated the active inference scheme for 16 time bins (196 ms) and then computed a map of salience to reset the prior expectations about the hidden control states that attract the center of gaze. Salience was computed for 1024 = 32 × 32 locations distributed uniformly over the visual image or scene. The prior expectation of the hidden control state was the location that maximized salience, according to Equation (28). The ensuing salience over the 32 × 32 locations constitutes a salience map that drives the next saccade. Notice that salience is a function of, and only of, fictive beliefs about the state of the world and essentially tells the agent where to look next.

**Figure 5:**
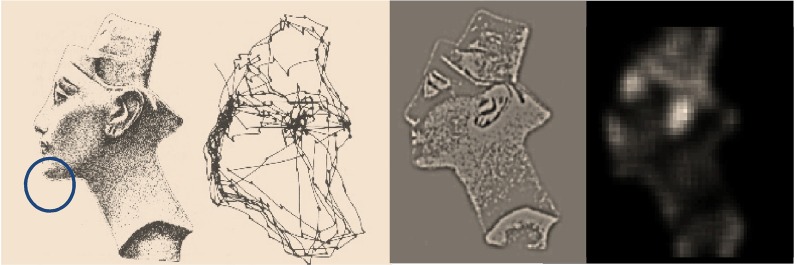
**This provides a simple illustration of salience based upon the posterior beliefs or hypothesis that local (foveal) visual inputs are caused by an image of Nefertiti.** The **left panels** summaries the classic results of the Yarbus; in terms of a stimulus and the eye movements it elicits. The **right panels** depict visual input after sampling the image on the right (using conventional center surround receptive fields) and the associated saliency map based on a local sampling of 16 × 16 pixels, using the generative model described in the main text. The size of the resulting field of view, in relation to the visual scene, is indicated with the circle on the left image. The key thing to note here is that the salient features of the image include the ear, eye, and mouth. The location of these features and other salient locations appear to be consistent with the locations that attract saccadic eye movements (as shown on the right).

Figure 5 provides a simple illustration of salience based upon the posterior beliefs or hypothesis that local (foveal) visual inputs are caused by an image of Nefertiti. The left panels summaries the classic results of the Yarbus (1967); in terms of a stimulus and the eye movements it elicits. The right panels depict visual input after sampling the image on the right with center-surround receptive fields and the associated saliency map based on a local sampling of 16 × 16 pixels, using Equation (21). Note how the receptive fields suppress absolute levels of luminance contrast and highlight edges. It is these edges that inform posterior beliefs about the content of the visual scene and where it is being sampled. This information reduces conditional uncertainty and is therefore salient. The salient features of the image include the ear, eye, and mouth. The location of these features and a number of other salient locations appear to be consistent with the locations that attract saccadic eye movements (as shown on the right). Crucially, the map of salience extends well beyond the field of view (circle on the picture). This reflects the fact that salience is not an attribute of what is seen, but what might be seen under a particular hypothesis about the causes of sensations.

To make the simulations a bit more realistic, we added a further prior implementing inhibition of return (Itti and Koch, 2001; Wang and Klein, 2010). This involved suppressing the salience of locations that have been recently foveated, using the following scheme:

(32) $$\begin{array}{l} S_{k}=S_{k}-(S_{k}\times R_{k-1})\\ R_{k}=\rho (S_{k})+\frac{1}{2}R_{k-1} \end{array}$$

Here, $S_{k}=S({\tilde{\eta }}_{j})-\min(S({\tilde{\eta }}_{j}))$ is the differential salience for the *k*-th saccade and *R*_*k*_ is an inhibition of return map that remembers recently foveated locations. This map reduces the salience of previous locations if they were visited recently. The function ρ(**S**_*k*_) ∈ [0, 1] is a Gaussian function (with a standard deviation of 1/16 of the image size) of the distance from the location of maximum salience that attracts the *k*-th saccade. The addition of inhibition of return ensures that a new location is selected by each saccade and can be motivated ethologically by prior beliefs that the visual scene will change and that previous locations should be revisited.

### Functional anatomy

**Figure 6:**
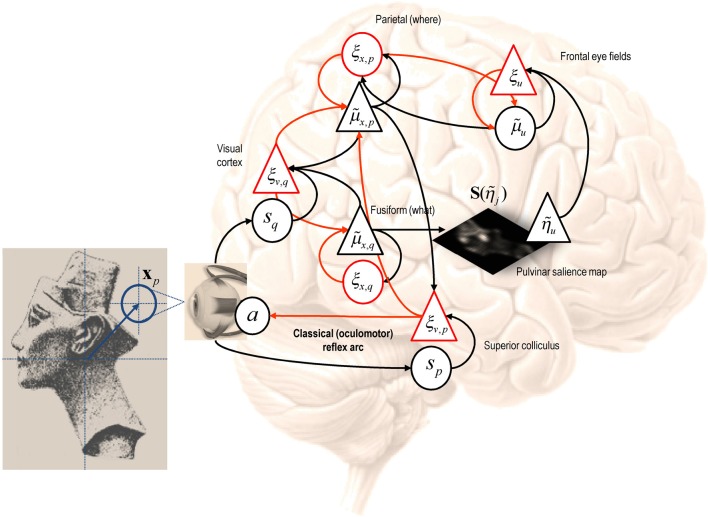
**This schematic depicts a particular instance of the message passing scheme in Figure 4.** This example follows from the generative model of visual input described in the main text. The model prescribes a particular hierarchical form for generalized predictive coding; shown here in terms of state and error units (black and red, respectively) that have been assigned to different cortical or subcortical regions. The insert on the left shows a visual scene (a picture of Nefertiti) that can be sampled locally by foveating a particular point—the true hidden state of the world. The resulting visual input arrives in primary visual cortex to elicit prediction errors that are passed forward to what and where streams. State units in the “what” stream respond by adjusting their representations to provide better predictions based upon a discrete number of internal images or hypotheses. Crucially, the predictions of visual input depend upon posterior beliefs about the direction of gaze encoded by state units in the “where” stream. These conditional expectations are themselves informed by top–down prior beliefs about the direction of gaze that maximizes salience. The salience map shown in the center is updated between saccades based upon posterior beliefs about the content of the visual scene. Posterior beliefs about the content of the visual scene provide predictions of visual input and future hidden states subtending salience. Posterior beliefs about the direction of gaze are used to form predictions of visual input and provide proprioceptive predictions to the oculomotor system in the superior colliculus and pontine nuclei, to elaborate a proprioceptive prediction error. This prediction error drives the oculomotor system to fulfill posterior beliefs about where to look next. This can be regarded as an instance of the classical reflects arc, whose set point is determined by top–down proprioceptive predictions. The variables associated with each region are described in detail in the text, while the arrows connecting regions adopt same format as in Figure 4 (forward prediction error afferents in red and backward predictions in black).

Figure 6 provides an intuition as to how active inference under salience priors might be implemented in the brain. This schematic depicts a particular instance of the message passing scheme in Figure 4, based on the generative model above. This model prescribes a hierarchical form for generalized predictive coding; shown here in terms of state and error units (black and red, denoting deep and superficial pyramidal cell populations, respectively) that have been assigned to different cortical or subcortical regions. The insert on the left shows a visual scene (a picture of Nefertiti) that can be sampled locally by foveating a particular point—the true hidden state of the world. The resulting visual input arrives in primary visual cortex to elicit prediction errors that are passed forward to “what” and “where” streams (Ungerleider and Mishkin, 1982). State units in the “what” stream respond by adjusting their representations to provide better predictions based upon a discrete number of internal images or hypotheses. Crucially, the predictions of visual input depend upon posterior beliefs about the direction of gaze, encoded by the state units in the “where” stream (Bisley and Goldberg, 2010). These posterior expectations are themselves informed by top–down prior beliefs about the direction of gaze that maximizes salience. The salience map shown in the center is updated between saccades based upon conditional expectations about the content of the visual scene. Conditional beliefs about the direction of gaze provide proprioceptive predictions to the oculomotor system in the superior colliculus and pontine nuclei, to elaborate a proprioceptive prediction error (Grossberg et al., 1997; Shires et al., 2010; Shen et al., 2011). This prediction error drives the oculomotor system to fulfill posterior beliefs about where to look next. This can be regarded as an instance of the classical reflects arc, whose set point is determined by top–down proprioceptive predictions. The anatomical designations should not be taken seriously (for example, the salience map may be assembled in the pulvinar or frontal cortex and mapped to the deep layer of the superior colliculus). The important thing to take from this schematic is the functional logic implied by the anatomy that involves reciprocal message passing and nested loops in a hierarchical architecture that is not dissimilar to circuits in the real brain. In particular, note that representations of hidden perceptual states provide bilateral top–down projections to early visual system is (to predict visual input) and to the systems computing salience, which might involve the pulvinar of the thalamus (Wardak et al., 2011; Wurtz et al., 2011).

### Simulating saccadic eye movements

**Figure 7:**
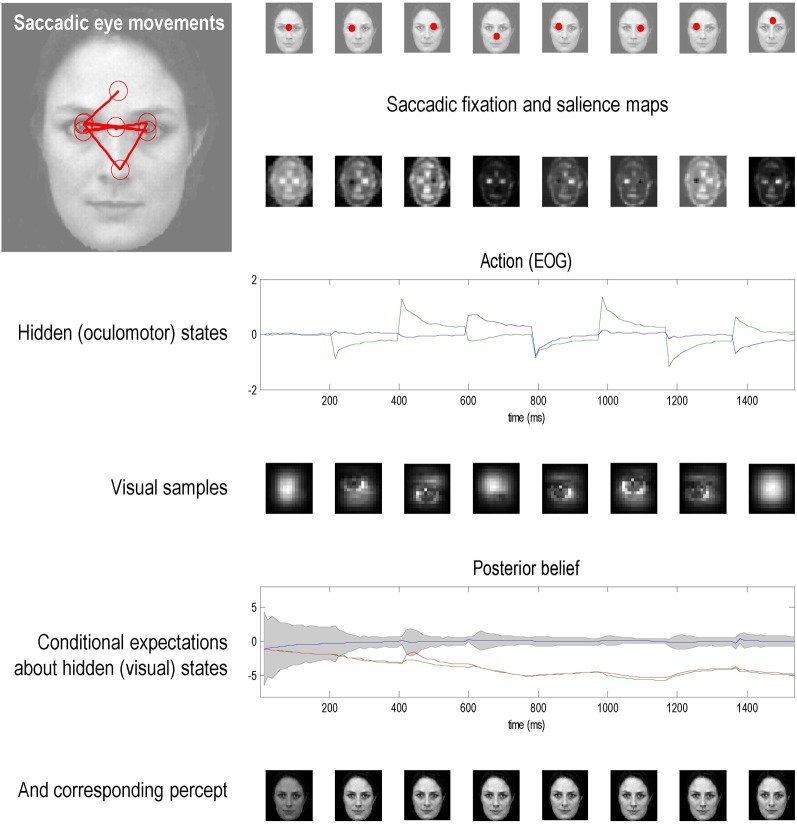
**This figure shows the results of a simulation, in which a face was presented to an agent, whose responses were simulated using the optimal inference scheme described in the main text.** In this simulation, the agent had three internal images or hypotheses about the stimuli it might sample (an upright face, an inverted face, and a rotated face). The agent was presented with an upright face and its conditional expectations were evaluated over 16 (12 ms) time bins until the next saccade was emitted. This was repeated for eight saccades. The ensuing eye movements are shown as red dots at the location (in extrinsic coordinates) at the end of each saccade in the upper row. The corresponding sequence of eye movements is shown in the insert on the upper left, where the red circles correspond roughly to the proportion of the image sampled. These saccades are driven by prior beliefs about the direction of gaze based upon the saliency maps in the second row. Note that these maps change with successive saccades as posterior beliefs about the hidden states, including the stimulus, become progressively more confident. Note also that salience is depleted in locations that were foveated in the previous saccade. These posterior beliefs provide both visual and proprioceptive predictions that suppress visual prediction errors and drive eye movements, respectively. Oculomotor responses are shown in the third row in terms of the two hidden oculomotor states corresponding to vertical and horizontal displacements. The associated portions of the image sampled (at the end of each saccade) are shown in the fourth row. The final two rows show the posterior beliefs in terms of their sufficient statistics and the stimulus categories, respectively. The posterior beliefs are plotted here in terms of conditional expectations and the 90% confidence interval about the true stimulus. The key thing to note here is that the expectation about the true stimulus supervenes over its competing expectations and, as a result, conditional confidence about the stimulus category increases (the confidence intervals shrink to the expectation). This illustrates the nature of evidence accumulation when selecting a hypothesis or percept the best explains sensory data.

Figure 7 shows the results of a simulated visual search, in which the agent had three internal images or hypotheses about the scene it might sample (an upright face, an inverted face, and a rotated face). The agent was presented with an upright face and its posterior expectations were evaluated over 16 (12 ms) time bins, after which salience was evaluated. The agent then emitted a saccade by foveating the most salient location during the subsequent 16 time bins—from its starting location (the center of the visual field). This was repeated for eight saccades. The upper row shows the ensuing eye movements as red dots (in the extrinsic coordinates of the true scene) at the fixation point of each saccade. The corresponding sequence of eye movements are shown in the insert on the upper left, where the red circles correspond roughly to the agent's field of view. These saccades were driven by prior beliefs about the direction of gaze based upon the salience maps in the second row. Note that these maps change with successive saccades as posterior beliefs about the hidden perceptual states become progressively more confident. Note also that salience is depleted in locations that were foveated in the previous saccade—this reflects the inhibition of return. Posterior beliefs about hidden states provide visual and proprioceptive predictions that suppress visual prediction errors and drive eye movements, respectively. Oculomotor responses are shown in the third row in terms of the two hidden oculomotor states corresponding to vertical and horizontal displacements. The portions of the image sampled (at the end of each saccade) are shown in the fourth row (weighted by the Hamming function above). The final two rows show the posterior beliefs in terms of their sufficient statistics (penultimate row) and the perceptual categories (last row), respectively. The posterior beliefs are plotted here in terms of posterior expectations and 90% confidence interval about the true stimulus. The key thing to note here is that the expectation about the true stimulus supervenes over its competing representations and, as a result, posterior confidence about the stimulus category increases (the posterior confidence intervals shrink to the expectation): see Churchland et al. (2011) for an empirical study of this sort phenomena. The images in the lower row depict the hypothesis selected; their intensity has been scaled to reflect conditional uncertainty, using the entropy (average uncertainty) of the softmax probabilities.

This simulation illustrates a number of key points. First, it illustrates the nature of evidence accumulation in selecting a hypothesis or percept the best explains sensory data. One can see that this proceeds over two timescales; both within and between saccades. Within-saccade accumulation is evident even during the initial fixation, with further stepwise decreases in uncertainty as salient information is sampled. The within-saccade accumulation is formally related to evidence accumulation as described in models of perceptual discrimination (Gold and Shadlen, 2003; Churchland et al., 2011). This is reflected in the progressive elevation of the correct perceptual state above its competitors and the consequent shrinking of the posterior confidence interval. The transient changes in the posterior beliefs, shortly after each saccade, reflect the fact that new data are being generated as the eye sweeps toward its new target location. It is important to note that the agent is not just predicting visual contrast, but also how contrast changes with eye movements—this induces an increase in conditional uncertainty (in generalized coordinates of motion) during the fast phase of the saccade. However, due to the veracity of the posterior beliefs, the conditional confidence shrinks again when the saccade reaches its target location. This shrinkage is usually to a smaller level than in the previous saccade.

This illustrates the second key point; namely, the circular causality that lies behind perception. Put simply, the only hypothesis that can endure over successive saccades is the one that correctly predicts the salient features that are sampled. This sampling depends upon action or an embodied inference that speaks directly to the notion of active vision or visual palpation (O'Regan and Noë, 2001; Wurtz et al., 2011). This means that the hypothesis prescribes its own verification and can only survive if it is a correct representation of the world. If its salient features are not discovered, it will be discarded in favor of a better hypothesis. This provides a nice perspective on perception as hypothesis testing, where the emphasis is on the selective processes that underlie sequential testing. This is particularly pertinent when hypotheses can make predictions that are more extensive than the data available at any one time.

Finally, although the majority of saccades target the eyes and nose, as one might expect, there is one saccade to the forehead. This is somewhat paradoxical, because the forehead contains no edges and cannot increase posterior confidence about a face. However, this region is highly informative under the remaining two hypotheses (corresponding to the location of the nose in the inverted face and the left eye in the rotated face). This subliminal salience is revealed through inhibition of return and reflects the fact that the two competing hypotheses have not been completely excluded. This illustrates the competitive nature of perceptual selection induced by inhibition of return and can regarded, heuristically, as occasional checking of alternative hypotheses. This is a bit like a scientist who tries to refute his hypothesis by acquiring data that furnish efficient tests of his competing or null hypotheses.

## Conclusion

This ideas reviewed in this paper suggest that the reward or cost-functions that underlie value in conventional (normative) models of optimal control can be cast as prior beliefs about future states, which are disclosed through active inference. In this setting, value becomes the evidence for generative models of our world—and valuable behavior is nothing more or less than accumulating evidence for our embodied models, through Bayesian updating of posterior beliefs. Subsequently, we saw that prior beliefs about future states are simply those that minimize the uncertainty of posterior beliefs. In this general formulation, we can understand exploration of the sensorium in terms of optimality principles based on ergodic or homoeostatic principles. In other words, to maintain the constancy of our external milieu, it is sufficient to expose ourselves to predicted and predictable stimuli. Being able to predict current observations also enables us to predict fictive sensations that we could experience from another viewpoint; where the best viewpoint is the one that confirms our predictions with the greatest precision or certainty. In short, action fulfills our predictions, while we predict the consequences of our actions will minimize uncertainty about those predictions. This provides a principled way in which to sample the world; for example, with visual searches using saccadic eye movements. These theoretical considerations are remarkably consistent with a number of compelling heuristics; most notably the Infomax principle or the principle of minimum redundancy, signal detection theory and formulations of salience in terms of Bayesian surprise.

An interesting perspective on active inference and embodied perception emerges from these considerations, in which percepts are selected through a form of circular causality: in other words, only the correct perceptual hypothesis can survive the cycle of action and perception, when the percept is used to predict where to look next. If the true state of the world and the current hypothesis concur, then the percept can maintain itself by selectively sampling evidence for its own existence. This provides an embodied (enactivist) explanation for perception that fits comfortably with the notion of visual sniffing or palpation (O'Regan and Noë, 2001; Wurtz et al., 2011). Furthermore, it resonates with neurodynamic accounts of self-generated behavior in a robotics context (Namikawa et al., 2011).

The arguments in this paper have been inspired by developments in theoretical neurobiology and machine learning. However, it is interesting to consider parallel developments in neurorobotics. Two decades ago most neurorobotics employed simple architectures with sensory-motor mappings implemented by perceptron-type networks and supervised learning; for example, the supervised learning of driving skills in robot cars (Pomerleau, 1991). In principle, active inference provides a formalism to revisit these sorts of problems using self-supervised schemes based upon deep hierarchical models. The usefulness of hierarchical schemes has been demonstrated by Morimoto and Doya, who show how a robot can stand up using hierarchical reinforcement learning (Morimoto and Doya, 2001). Furthermore, the idea of forward (predictive) modeling is now established in neurorobotics: Schaal (1997) has shown how learning a predictive forward model is beneficial in imitation learning, while Tani and Nolfi (1999) show how prediction error can be used to recognize self-generated behavior using a hierarchically organized mixture of predictive expert networks. There are clear parallels here with active inference under hierarchical generative (forward) models that suggest a theoretical convergence of neurobiology and neurorobotics. One can imagine exploiting the fairly simple and principled optimization schemes provided by free energy minimization to elaborate robots with deep hierarchical models, were these models that generally entail a separation of temporal scales and context sensitive behavior. On a more general note, active inference may provide a formal framework that connects the compelling work in neurorobotics on imitation and action observation to some of the highest level questions that currently preoccupy psychologists and cognitive neuroscientists—particularly those people interested in psychopathology and its mechanistic underpinnings.

The treatment of optimality in this paper has focused on the nature of value and its relationship to evidence. There are many other important issues that we have glossed over; such as the acquisition or learning of models. For example, as noted by one of our reviewers: “Many traditional (alternate) methods would be capable of arriving at optimal policies despite limitations in the model, owing to the properties of the approximation procedures. In the authors' proposal, the underlying generative model would need to capture the necessary dynamics through the definition of the priors and model structure (which the authors note may be learnt separately at a higher level). Do we know that this internal model can be learnt, in a tractable form given what can be known about the task? Do we know if the solutions to the two cases will be similar?”

In one sense, traditional methods are not necessarily alternative methods, because optimal policies can be cast as prior beliefs. In other words, the current framework just allows one to convert optimal control problems into pure inference problems. The motivation for this is to understand where prior beliefs (optimal policies) come from in a hierarchical setting. The hierarchical aspect is important because this necessarily induces empirical priors, which means that cost functions can themselves be optimized in relation to model evidence. This is illustrated nicely in the context of learning and model selection: a fuller treatment would show that the parameters of any given model can be learned in a Bayes optimal fashion by minimizing variational free energy (Friston, 2008). Furthermore, the model itself can also be optimized with respect to variational free energy, in exactly the same way that Bayesian model selection operates in data analysis. This hierarchical optimization may provide a nice metaphor for understanding selection at a neurodevelopmental or evolutionary timescale (Friston et al., 2006). Crucially, because we are dealing with approximate Bayesian inference, the models selected will necessarily be approximations and provide the simplest (most parsimonious) explanations for sampled outcomes. In answer to the reviewer's questions, any extant phenotype is an existence proof that its particular (approximate) model can be learnt. The question about the uniqueness of models is a bit more subtle—in the sense that (in active inference) models create their own data. This means that each phenotype may be a uniquely optimal model for its own sensorium but not that of another phenotype. These are clearly very important issues, which motivate the work reviewed in this paper.

The ideas described in this paper try to go beyond the formal similarity between optimal control and Bayesian inference schemes to suggest that optimal control is a special case of Bayes-optimal inference and that inference is the hard problem. In this setting, optimality reduces to sampling states prescribed by the priors of a generative model that specifies state transitions. So what are the practical advantages of casting optimal control as inference? In Friston et al. (2012b) we summarized the advantages of active inference as providing:

- A tractable approximate solution to any stochastic, non-linear optimal control problem to the extent that standard (variational) Bayesian procedures exist. Variational or approximate Bayesian inference is well-established in statistics and data assimilation because it finesses many of the computational problems associated with exact Bayesian inference.
- The opportunity to learn and infer environmental constraints in a Bayes-optimal fashion; particularly the parameters of equations of motion and amplitudes of observation and hidden state noise.
- The formalism to handle system or state noise: currently, optimal control schemes are restricted to stochastic control (i.e., random fluctuations on control as opposed to hidden states). One of the practical advantages of active inference is that fluctuations in hidden states are modeled explicitly, rendering control robust to exogenous perturbations.
- The specification of control costs in terms of priors on control, with an arbitrary form: currently, most approximate stochastic optimal control schemes are restricted to quadratic control costs. In classical schemes that appeal to path integral solutions there are additional constraints that require control costs to be a function of the precision of control noise; e.g., Theodorou et al. (2010) and Braun et al. (2011). These constraints are not necessary in active inference.

The disadvantage of active inference is that one cannot prescribe optimality in terms of cost functions, because (Bayes) optimal behavior rests on a generative model that is specified by its likelihood and prior functions. Having said this, for every Bayes-optimal policy there is an associated cost function (Friston and Ao, 2012). Perhaps the most important advantage of active inference—for practical applications—is its simplicity and robustness. It simplicity stems from the fact that one only has to specify desired movements or trajectories in terms of prior beliefs (equations of motion in the generative model) as opposed to desired endpoints of movement (which requires the solution of a generally intractable optimal control problem). The robustness follows from the context sensitivity of active inference schemes and their ability to handle unpredicted (random) fluctuations or indeed changes in the motor plant—see Friston et al. (2010b). Finally, treating control problems as inference problems allows one to exploit the advances made in approximate Bayesian inference and model selection. A nice example here would be the hierarchal optimization of control architectures using Bayesian model selection and free energy as an approximation to log model evidence. This strategy is now used routinely to select among thousands of models within a few seconds (Friston and Penny, 2011) but has only been applied in a data analysis setting. In principle, these Bayesian procedures could also be used in a control setting.

In summary, we have tried to formalize the intuitive notion that our interactions with the world are akin to sensory experiments, by which we confirm our hypotheses about its causal structure in an optimal and efficient fashion. This mandates prior beliefs that the deployment of sensory epithelia and our physical relationship to the world will disclose its secrets—beliefs that are fulfilled by action. The resulting active or embodied inference means that not only can we regard perception as hypothesis testing, but we could regard action as performing experiments that confirm or disconfirm those hypotheses.

### Conflict of interest statement

The authors declare that the research was conducted in the absence of any commercial or financial relationships that could be construed as a potential conflict of interest.
